# Body-mass index, blood pressure, diabetes and cardiovascular mortality in Cuba: prospective study of 146,556 participants

**DOI:** 10.1186/s12889-021-10911-9

**Published:** 2021-05-27

**Authors:** Nurys B Armas Rojas, Ben Lacey, Monica Soni, Shaquille Charles, Jennifer Carter, Patricia Varona-Pérez, Julie Ann Burrett, Marcy Calderón Martínez, Elba Lorenzo-Vázquez, Sonia Bess Constantén, Hannah Taylor, Paul Sherliker, José Manuel Morales Rigau, Stephanie Ross, M. Sofia Massa, Osvaldo Jesús Hernández López, Nazrul Islam, Miguel Ángel Martínez Morales, Ismell Alonso Alomá, Fernando Achiong Estupiñan, Mayda Díaz González, Noel Rosquete Muñoz, Marelis Cendra Asencio, Oscar Díaz-Diaz, Ileydis Iglesias-Marichal, Jonathan Emberson, Richard Peto, Sarah Lewington

**Affiliations:** 1 National Institute of Cardiology and Cardiovascular Surgery, Havana, Cuba; 2 Nuffield Department of Population Health (NDPH), University of Oxford, Oxford, UK; 3 Georgetown University School of Medicine, Washington, D.C, USA; 4 University of Pittsburgh School of Medicine, Pittsburgh, PA USA; 5 Institute of Hygiene, Epidemiology and Microbiology, Ministry of Public Health, Havana, Cuba; 6 Cuban Commission against Smoking, Ministry of Public Health, Havana, Cuba; 7 Directorate of Medical Records and Health Statistics, Ministry of Public Health, Havana, Cuba; 8 MRC Population Health Research Unit, NDPH, University of Oxford, Oxford, UK; 9 Provincial Center of Hygiene, Epidemiology and Microbiology, Matanzas, Cuba; 10 Municipal Center of Hygiene, Epidemiology and Microbiology, Jagüey Grande, Matanzas, Cuba; 11 Municipal Center of Hygiene, Epidemiology and Microbiology, Colón, Matanzas, Cuba; 12 Municipal Center of Hygiene, Epidemiology and Microbiology, Camagüey, Cuba; 13 National Institute of Endocrinology, Havana, Cuba; 14 UKM Medical Molecular Biology Institute (UMBI), Universiti Kebangsaan Malaysia, Kuala Lumpur, Malaysia

**Keywords:** Cuba, Cardiovascular, Body-mass index, Blood pressure, Diabetes

## Abstract

**Background:**

Cardiovascular disease accounts for about one-third of all premature deaths (ie, age < 70) in Cuba. Yet, the relevance of major risk factors, including systolic blood pressure (SBP), diabetes, and body-mass index (BMI), to cardiovascular mortality in this population remains unclear.

**Methods:**

In 1996–2002, 146,556 adults were recruited from the general population in five areas of Cuba. Participants were interviewed, measured (height, weight and blood pressure) and followed up by electronic linkage to national death registries until Jan 1, 2017; in 2006–08, 24,345 participants were resurveyed. After excluding all with missing data, cardiovascular disease at recruitment, and those who died in the first 5 years, Cox regression (adjusted for age, sex, education, smoking, alcohol and, where appropriate, BMI) was used to relate cardiovascular mortality rate ratios (RRs) at ages 35–79 years to SBP, diabetes and BMI; RR were corrected for regression dilution to give associations with long-term average (ie, ‘usual’) levels of SBP and BMI.

**Results:**

After exclusions, there were 125,939 participants (mean age 53 [SD12]; 55% women). Mean SBP was 124 mmHg (SD15), 5% had diabetes, and mean BMI was 24.2 kg/m^2^ (SD3.6); mean SBP and diabetes prevalence at recruitment were both strongly related to BMI. During follow-up, there were 4112 cardiovascular deaths (2032 ischaemic heart disease, 832 stroke, and 1248 other). Cardiovascular mortality was positively associated with SBP (>=120 mmHg), diabetes, and BMI (>=22.5 kg/m^2^): 20 mmHg higher usual SBP about doubled cardiovascular mortality (RR 2.02, 95%CI 1.88–2.18]), as did diabetes (2.15, 1.95–2.37), and 10 kg/m^2^ higher usual BMI (1.92, 1.64–2.25). RR were similar in men and in women. The association with BMI and cardiovascular mortality was almost completely attenuated following adjustment for the mediating effect of SBP. Elevated SBP (>=120 mmHg), diabetes and raised BMI (>=22.5 kg/m^2^) accounted for 27%, 14%, and 16% of cardiovascular deaths, respectively.

**Conclusions:**

This large prospective study provides direct evidence for the effects of these major risk factors on cardiovascular mortality in Cuba. Despite comparatively low levels of these risk factors by international standards, the strength of their association with cardiovascular death means they nevertheless exert a substantial impact on premature mortality in Cuba.

**Supplementary Information:**

The online version contains supplementary material available at 10.1186/s12889-021-10911-9.

## Background

In 2013, the WHO World Health Assembly adopted the global goal of a 25% relative reduction in premature mortality (before age 70 years) from non-communicable diseases (NCDs) through nine voluntary targets by 2025 [1]. Cardiovascular disease (including heart disease, stroke and peripheral vascular disease) accounts for about half of all premature NCD deaths globally [2], and several of the WHO voluntary targets were aimed specifically at cardiovascular mortality, including those to reduce the prevalence of raised blood pressure, and to halt the rising prevalence of diabetes and obesity.

However, the expected benefit on cardiovascular mortality from addressing these risk factors in different populations is based mainly on prospective studies in high-income countries [3–7]. There is a paucity of large prospective studies conducted in other parts of the world. For example, a meta-analysis of 239 prospective studies from the Global BMI Mortality Collaboration does not include any studies from Latin America [5]. Furthermore, recent findings from one of the few large studies in this region, the Mexico City Prospective Study, indicates that the effect of some of these risk factors may be very different from that in high-income countries. In particular, that study found that those with diabetes had about four times the death rate from cardiovascular disease compared with people without diabetes [8]; in high-income countries, diabetes about doubles the cardiovascular death rate [6].

Cuba is a middle-income country, with a population of 11.3 million in 2020 [9]. The health system is focused particularly on primary care and disease prevention, which has delivered substantial reductions in infant mortality over the last few decades but death rates in middle age remain high [10]. Cardiovascular disease accounts for one-third of all premature deaths in Cuba [11], yet substantial uncertainty remains about the relevance of major metabolic risk factors to cardiovascular mortality in this population. We report the associations of blood pressure, diabetes and body-mass index (BMI) with cardiovascular mortality in a large, population-based prospective study of 146,556 adults in Cuba.

## Methods

### Study design and participants

In 1996–2002, 146,556 adults aged ≥30 years were recruited from the general population in Cuba into a prospective cohort study. Details of the study design and survey methods have been reported previously [11]. Briefly, the study recruited men and women from five of Cuba’s fourteen provinces. Within each province, medical offices were selected at random using a computer-generated random allocation sequence. Each medical office subsequently invited all of its patients aged 30 years and older to participate in the study. Overall, 74% of eligible individuals participated.

Trained health-care staff (mostly the family doctor, but on occasion local nurses or other trained health care workers) visited each household. After providing written consent, participants provided information on age, sex, ethnicity, education, occupation, marital status, lifestyle factors, current medications, and medical history (the original questionnaire in Spanish, developed solely for this study, and the English translation are available in Additional file 1: p2–3). Blood pressure was measured twice while the participant was seated (once towards the beginning of the interview and once towards the end), using a calibrated manual sphygmomanometer and standard techniques. Following the home visit, participants were invited to attend their physician’s medical office for height and weight measurements. Between 2006 and 2008, baseline measures were repeated using the same procedures in a formal resurvey of 24,345 participants (17% of the initial study population; all residents of Matanzas province) to assess temporal variation in estimated levels of risk factors at baseline, particularly blood pressure and BMI.

Participants were followed up until 1st January 2017 through electronic record linkage to the Cuban Public Health Ministry’s mortality records using national identification numbers. Mortality records are collected for all deaths in Cuba, and include medically certified causes of death. In 1996–2000, all deaths in Cuba were coded using the 9th edition of the International Classification of Disease (ICD-9), while all deaths from 2001 onwards were coded using ICD-10 (Additional file 1: Table S1).

### Statistical analyses

The presence of diabetes was defined as a (self-reported) previous medical diagnosis of diabetes, the use of antidiabetic medication (oral hypoglycemic medication or insulin), or both. The mean of the two systolic blood pressure (SBP), or the two diastolic blood pressure (DBP), measurements was used in all blood pressure analyses, and body-mass index (BMI) was calculated as weight in kg divided by the square of height in m.

Participants with missing SBP, DBP, diabetes status, BMI or other key covariates, were excluded from the main analyses, as were those with implausible or outlying values for SBP (<80 or ≥250 mmHg), DBP (<40 or ≥ 150mmHg), or BMI (<15 or ≥40 kg/m^2^), those with prior cardiovascular disease at baseline (myocardial infarction, angina, or stroke), and those with no follow-up at ages 35–79 years. To further limit any effect of pre-existing disease on blood pressure or BMI at baseline, the first 5 years of follow-up were also excluded.

Cox regression was used to relate mortality rate ratios (RR) at ages 35–79 years to SBP (using the mean of the two SBP measurements), diabetes and BMI, with adjustment for age at risk (5-year groups), sex, highest completed level of formal education (less than primary, primary, lower secondary, high school [or technical training], university), smoking (never, ex-smoker, current smoker of < 20 cigarettes/day, current smoker of 20 cigarettes/day, current smoker of >20 cigarettes/day, other smoker), alcohol (non- drinker, less than weekly drinker, at least weekly drink of < 1 bottle/week of 35 cl rum [or equivalent alcohol] per week, at least weekly drinker of 1- <3 bottles/week, and at least weekly drinker of 3 plus bottles/week), and, where appropriate, BMI (15- <20, 20- <22.5, 22.5- <25, 25- <27.5, 27.5- <30, 30- <40 kg/m^2^); analyses of DBP are given in Additional file 1. RRs were corrected for regression dilution (ie, categorising people by their baseline SBP or BMI and estimating the long-term average mean SBP or BMI in each category using the correlation between re-survey and baseline measurements [12]), and are therefore described as associations of *usual* SBP and *usual* BMI with mortality [3, 4].

In categorical analyses, SBP was categorized as <125, 125- <145, 145- <165, ≥165 mmHg, and BMI was categorized as 15- <20, 20- <22.5, 22.5- <25, 25- <27.5, 27.5- <30, 30- <35, 35- <40 kg/m^2^. Confidence intervals (CIs) were calculated using the variance of the log risk, which appropriately attributes variance to all groups, including the reference, and so allows CIs to be used to compare risks in any two groups [13]. Linear associations are reported per 20 mmHg higher usual SBP and per 10 kg/m^2^ higher usual BMI.

Sensitivity analyses of the main prospective associations were conducted by further adjusting the associations for other potential confounders, and by excluding participants with non-vascular chronic diseases at baseline (including chronic obstructive pulmonary disease, liver cirrhosis, chronic kidney disease, peptic ulcer, and cancer) and those taking blood pressure-lowering medication. The analyses of BMI are also reported with and without further adjustment for usual SBP and diabetes (including the effect of such adjustments on the Wald chi-squared statistic), to assess the extent to which these factors mediate the association.

The fraction of cardiovascular deaths in Cuba attributed to SBP, diabetes and BMI were estimated by age group and sex, using the formula P_e_ (RR – 1)/(P_e_ [RR-1] + 1) where P_e_ is the prevalence of the given risk factor in Cuba as estimated by the 2010 Cuban National Non-communicable Disease Risk Factor Survey [14], and RR is the rate ratio in the present study [15]. These fractions were multiplied by the number of vascular deaths in each age group and sex in Cuba in 2015 [16] to give the overall number of deaths attributable to each of these risk factors at ages 35–79 years. All analyses were conducted in SAS (version 9.4), and results were plotted in R (version 3.6.2).

## Results

Of 146,556 adults recruited into the study, 4941 were excluded because of missing or extreme values, 10,053 were excluded because they had prior cardiovascular disease and 7687 with no follow-up at ages 35 to 79 years, leaving 125,939 participants (exclusion categories were not mutually exclusive, see Additional file 1: Table S2).

After exclusions, the mean age of participants was 53 years (SD 12), 55% were female, and 33% had received no formal education (Table 1). Mean SBP was 124 mmHg (SD 15), mean BMI was 24.2 kg/m^2^ (SD 3.6), and 5% of participants had prior diabetes. There was little difference in mean SBP and mean BMI by sex, but diabetes prevalence was about twice as high in women than in men (7% vs 4%; Additional file 1: Table S3).

**Table 1:**
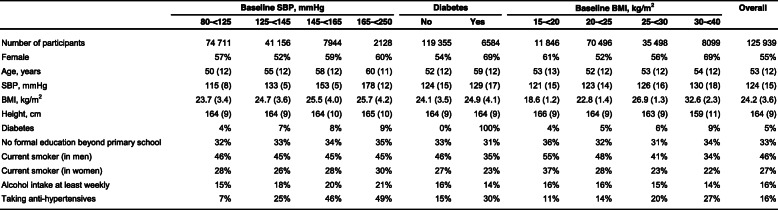
Baseline characteristics, by systolic blood pressure (SBP), diabetes and BMI at baseline. Data are % or mean (SD). Results are standardised to the age and sex of the 125 939 participants. Participants with no follow-up at ages 35-79 years, those with pre-existing vascular disease at baseline, and those with missing or outlying values for SBP, diabetes, BMI or key covariates were excluded. Baseline characteristics by four groups of BMI are given for clarity, but are given by 7 groups in Appendix, Table S4

**Fig. 1:**
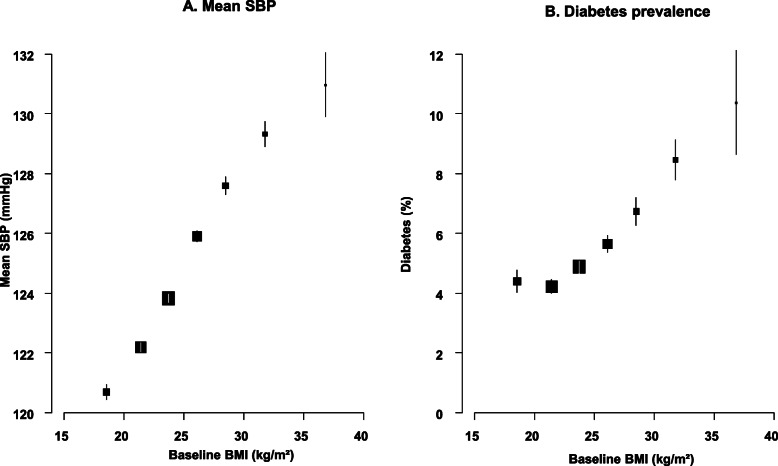
Baseline associations of mean systolic blood pressure (SBP) and diabetes prevalence vs BMI. Results are standardised to the age and sex of the 125,939 participants; exclusions as in Table 1. For each category, area of the square is inversely proportional to the variance of the mean SBP, or the prevalence of diabetes, which also determines the confidence interval (CI)

At baseline, SBP was positively correlated with mean BMI, diabetes and at least weekly drinking, but was not strongly related to smoking (Table 1). On average, those with diabetes were more likely to be female, older and with higher SBP and BMI, but with lower prevalence of weekly drinking and smoking. BMI was inversely related to smoking and drinking (Additional file 1: Table S4), and there was an approximately linear association of BMI with SBP (~ 1 mmHg per kg/m^2^) and with diabetes prevalence in the range > 20 kg/m^2^ BMI (~ 1% per kg/m^2^: Table 1, Fig. 1).

At resurvey, mean SBP at ages 40–79 years (age-standardised to match the baseline distribution) was 2 mmHg lower than at baseline, diabetes prevalence was 3% higher, and mean BMI was 0.5 kg/m^2^ higher (Additional file 1: Table S5). The self-correlations between baseline and resurvey measurements (used to correct for regression dilution bias) were 0.48 for SBP and 0.59 for BMI (Additional file 1: Figure S1, Table S6).

After excluding the first 5 years of follow-up, there were 9987 deaths from all causes at ages 35–79 during 2.1 million person-years of follow-up (mean 17.6 [SD 3.0]): 4112 deaths were cardiovascular (2134 in men and 1978 in women), with 2032 deaths from ischaemic heart disease, 832 stroke, and 1248 other vascular.

**Fig. 2:**
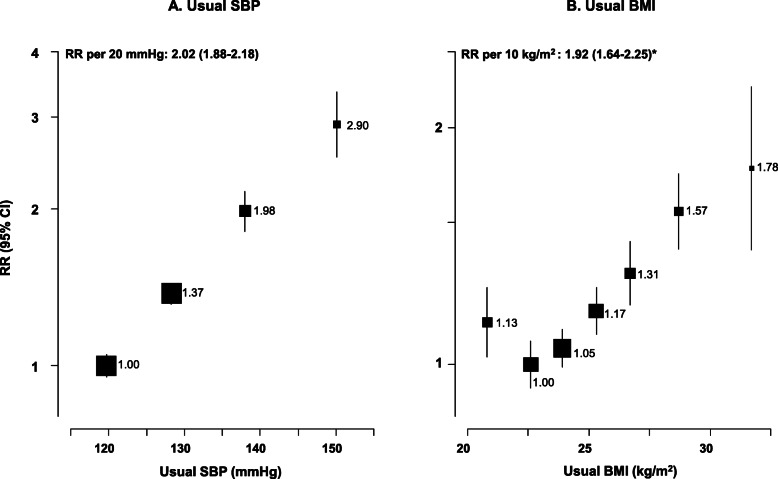
Vascular mortality vs usual systolic blood pressure (SBP) and usual BMI at ages 35–79 years in Cuba. Rate ratios (RR) adjusted for age, sex, education, province, smoking, alcohol, and BMI (when appropriate). Analyses omitted the first 5 years of follow up. Exclusions as in Table 1. For each SBP or BMI category, area of the square is inversely proportional to the variance of the category−specific log risk, which also determines the confidence interval (CI)

**Fig. 3:**
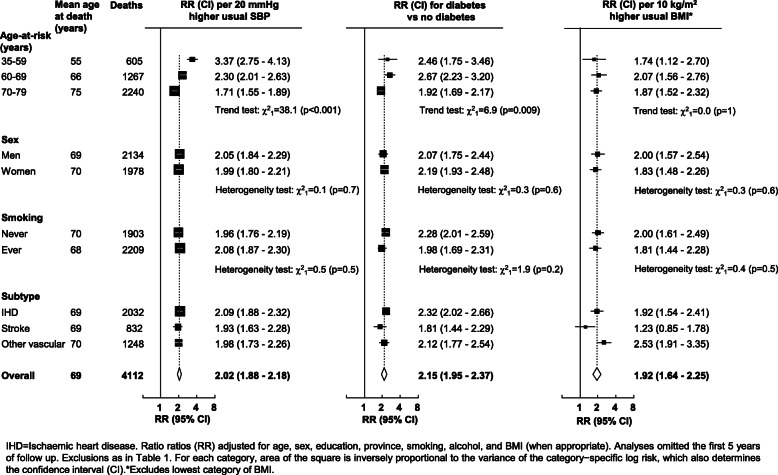
Death rate ratios (RR) for vascular mortality vs usual systolic blood pressure (SBP), diabetes and usual BMI at ages 35–79 years in Cuba. IHD=Ischaemic heart disease. Rate ratios (RR) adjusted for age, sex, education, province, smoking, alcohol, and BMI (when appropriate). Analyses omitted the first 5 years of follow up. Exclusions as in Table 1. For each category, area of the square is inversely proportional to the variance of the category−specific log risk, which also determines the confidence interval (CI).*Excludes lowest category of BMI

Usual SBP was strongly positively associated with cardiovascular mortality (Fig. 2) throughout the usual SBP range examined, with no evidence of a threshold down to at least 120 mmHg. Overall, each 20 mmHg higher usual SBP approximately doubled cardiovascular mortality (RR 2.02 [95% CI 1.88–2.18]; mean age at death 69: Fig. 3). The strength of the association was somewhat greater at younger than older ages, but there was no evidence that the strength of the association varied by sex or by smoking habits, or differed between major subtypes of cardiovascular mortality (ischaemic heart disease, stroke or other vascular) (Fig. 3). The association of cardiovascular mortality with usual DBP was also log-linear throughout the range examined (RR per 10 mmHg higher DBP of 1.75 [95% CI 1.63–1.87]; Additional file 1: Figure S2).

Previously diagnosed diabetes at baseline was associated with about twice the risk of cardiovascular mortality compared to those without diagnosed diabetes (RR 2.15 [95% CI 1.95–2.37]; Fig. 3). The strength of the association was slightly weaker at age 70–79 years than at younger ages (heterogeneity: χ_1_^2^ = 6.9; *p* = 0.009), but, as with usual SBP, the associations did not materially differ by sex, smoking or between major subtypes of cardiovascular death (Fig. 3; Additional file 1: Figure S3).

In analyses of cardiovascular mortality with usual BMI, mortality was lowest at about 22.5 kg/m^2^ (Fig. 2). There was a strong positive association above this level, and a slight inverse association below it. Above 22.5 kg/m^2^, each 10 kg/m^2^ higher usual BMI approximately doubled the risk of cardiovascular mortality (RR 1.92 [95% CI 1.64–2.25]). There was no evidence that the strength of this association varied by age, sex or smoking, but the association was somewhat shallower for stroke than for ischaemic heart disease or other vascular death (Fig. 3; Additional file 1: Figure S4).

**Fig. 4:**
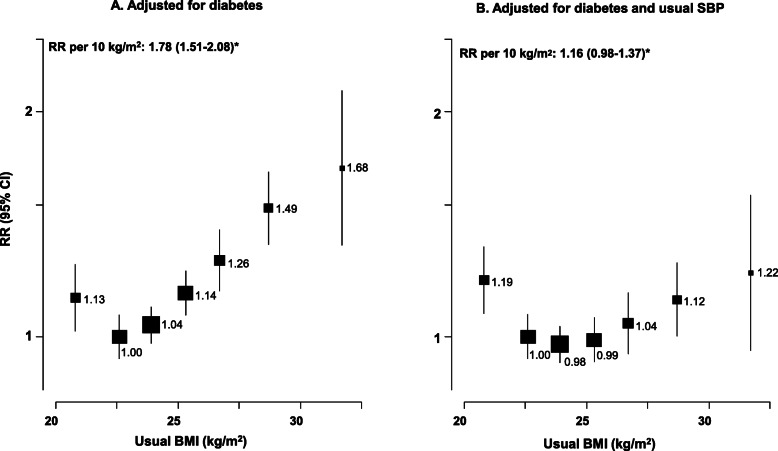
Vascular mortality vs usual BMI at ages 35–79 years in Cuba, with further adjustment for diabetes and usual systolic blood pressure (SBP). Rate ratios (RR) adjusted for age, sex, education, province, smoking, and alcohol, with further adjustment for diabetes and usual SBP when indicated. Analyses omitted the first 5 years of follow up. Exclusions as in Table 1. For each BMI category, area of the square is inversely proportional to the variance of the category−specific log risk, which also determines the confidence interval (CI). *Excludes lowest category of BMI

Figure 4 shows the association of usual BMI and cardiovascular mortality with further adjustment for diabetes and systolic blood pressure, both potential mediators of the association. The association was little changed following further adjustment for diabetes (the Wald chi-squared statistic for the model was reduced by 24% from 65 to 49). However, further adjustment for usual SBP almost completely attenuated the association: each 10 kg/m^2^ higher usual BMI in the range ≥ 22.5 kg/m^2^ (following adjustment for usual SBP) was associated with RR of 1.16 (95% CI 0.98–1.37; Wald X = 3.0).

In sensitivity analyses (Additional file 1: Figure S5-S10), further adjustment for other potential confounders did not materially change the associations; neither did exclusion of those with non-vascular chronic diseases at baseline (including chronic obstructive pulmonary disease, liver cirrhosis, chronic kidney disease, peptic ulcer, and cancer) or exclusion of those taking blood pressure-lowering medication. In additional analyses (Additional file 1: Figure S11) of the association of SBP, diabetes and BMI with cardiovascular deaths other than ischaemic heart disease or stroke, there were few deaths from any specific vascular diseases (such as hypertensive heart disease, heart failure, atherosclerosis, or aortic aneurysm), and little evidence of heterogeneity in the strength of these associations.

The excess cardiovascular mortality associated with elevated systolic blood pressure (ie, >=120 mmHg), diabetes and BMI (>=22.5 kg/m^2^) accounted for 27%, 14% and 16% of cardiovascular deaths in the age group 35–79 years (Table 2). These population-attributable fractions would be equivalent to around 5000 excess deaths due to elevated systolic blood pressure, 2600 excess deaths due to diabetes, and 3000 excess deaths due to raised BMI among people aged 35–79 years in Cuba in 2015.

**Table 2:**
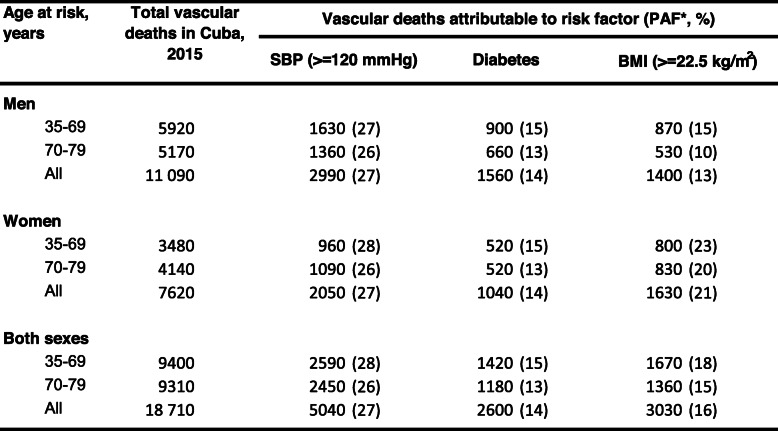
Vascular deaths attributable to systolic blood pressure (SBP), diabetes and BMI in Cuba, 2015. Vascular deaths (rounded to the nearest multiple of 10) attributed to each risk factor were calculated by applying population attributable fractions in the Cuba Prospective Study to the age- and sex-specific numberof vascular deaths in Cuba for 2015. *PAF=Population attributable fraction

## Discussion

In this large prospective study in Cuba, cardiovascular mortality was strongly positively associated with SBP, diabetes, and BMI. Overall, each 20 mmHg higher usual SBP approximately doubled cardiovascular mortality, as did prior diabetes, and 10 kg/m^2^ higher usual BMI (at ≥22.5 kg/m^2^). The strength of the associations were broadly similar in men and women, and across each of the major cardiovascular subtypes. The association of cardiovascular mortality with usual BMI was little changed following adjustment for diabetes (perhaps reflecting the low prevalence of diabetes in this population at baseline) but was almost completely attenuated following adjustment for SBP. About one-quarter of cardiovascular mortality was accounted for by elevated SBP, while diabetes and raised BMI each accounted for about 15% of cardiovascular mortality.

Few prospective studies have reported on the effect of these major risk factors on cardiovascular mortality in Latin America. In contrast to the present report, the Mexico City Prospective Study of 150,000 middle-aged adults found previously diagnosed diabetes at baseline was associated with about four times the death rate from cardiovascular disease compared with adults without diabetes [8]. The excess cardiovascular mortality associated with diabetes in the present report is more consistent with findings from prospective studies in high-income countries, where diabetes approximately doubles cardiovascular death rates [6]. However, unlike many studies in high-income countries, there was no evidence that the strength of the association with diabetes differed by sex (most studies have reported greater risks among women than men) [6, 17].

The association of cardiovascular mortality with SBP is also consistent with prospective studies in high-income countries. A meta-analysis of 61 prospective studies (conducted mostly in Europe or North America) found, at ages 40–69 years, 20 mmHg higher SBP approximately doubled cardiovascular mortality, with weaker associations at older ages [3]. As in the present report, there were log-linear associations with no evidence of a threshold down to low levels of blood pressure (at least 115 mmHg SBP and 75 mmHg DBP). However, in contrast to the findings in the present study, the proportional risks were stronger among men than women, particularly at younger ages.

Recent evidence suggests substantial variation in the relation between BMI and cardiovascular mortality in different parts of the world. In a meta-analysis of 239 prospective studies from the Global BMI Mortality Collaboration, both all-cause and cardiovascular mortality were lowest in the BMI range 20–<25 kg/m^2^ [5]. In the BMI range >=25 kg/m^2^, 5 kg/m^2^ higher BMI was associated with about 40% higher risk of cardiovascular mortality in North America (RR 1.38 [95% CI 1.32–1.44]), which is consistent with the present study (RR per 5 kg/m^2^ higher BMI of 1.39 [1.28–1.50]), but the strength of the associations was somewhat greater in studies from East Asia (RR 1.67 [1.50–1.85]), and there was little association in South Asian studies (although they included too few participants for statistical stability) [5]; the findings in South Asia were supported later by a prospective study of 0.5 million adults in South India [18].

This study has a number of key strengths. It is one of the largest studies to assess the effects of metabolic factors on cardiovascular mortality in Latin America. The study conducted a large resurvey to allow correction for regression dilution and reliable estimates of long-term average levels of SBP and BMI. The baseline survey collected information on prior disease to allow exclusion of those with prevalent cardiovascular disease, and on a range of factors to allow adjustment for major potential confounders. Furthermore, linkage to national mortality registers limited loss to follow-up and allowed reliable ascertainment of causes of deaths.

It is a limitation of the study that blood was not collected at baseline and as such it was not possible to identify those with undiagnosed diabetes. Neither was information collected on other measures of adiposity, such as waist circumference or body fat percentage; recent studies in mainly European populations indicate waist circumference, in particular, is associated with risk of vascular disease independently of BMI or other anthropometric measures (such as hip circumference), suggesting that BMI alone may underestimate the relevance of adiposity to vascular mortality [19, 20]. It was also not possible to further sub-classify cardiovascular outcomes, such as ischaemic and haemorrhagic stroke, which may have different relationships with the risk factors examined [21]. Indeed, a number of studies have reported a strong positive association of BMI with ischaemic stroke but little association with haemorrhagic stroke [22, 23]. In addition, as the baseline survey was conducted following a period of particular economic hardship (commonly referred to as the ‘Special Period’ in Cuba), the prevalence of the risk factors in the cohort is likely to have changed over the period of follow-up. However, steps were taken to limit the effect of such changes, including the correction for temporal variation of these factors in prospective analyses, and use of risk factors’ prevalence estimates from the 2010 Cuban National Non-communicable Disease Risk Factor Survey when calculating attributable fractions.

The health system in Cuba focuses particularly on disease prevention and primary care [10]. Primary care practices (normally consisting of a family doctor and nurse) are assigned to look after about 150–200 families, and healthcare staff live close to the communities they serve. Practices are encouraged to visit families and address major risk factors for common diseases through both advice on lifestyle modification and medication; an approach that might account for the lower cardiovascular mortality rates among diabetics in Cuba than Mexico, where poor glycaemic control and low use of cardioprotective medications may account for their poorer prognosis. The model of healthcare in Cuba has delivered substantial improvements in infant and maternal mortality over the last few decades, but mortality in middle age remains high with risk of death between ages 35–69 years in 2015 of 25% in men and 17% in women [16].

The findings in the present report will support efforts to address major metabolic risk factors for cardiovascular disease in Cuba. In particular, here, as elsewhere, the study confirms the importance of blood pressure to cardiovascular mortality. Trials have found blood pressure-lowering medication reduces the risk of both coronary heart disease and stroke [24], and the magnitude of the effect is consistent with findings on blood pressure and cardiovascular disease from prospective studies. The baseline survey, and more recent national surveys in Cuba [14], suggest that many with elevated blood pressure and at high absolute risk of cardiovascular mortality are not being treated with antihypertensives, and only about a third of treated patients have controlled blood pressure (defined as <140 mmHg SBP and <90 mmHg DBP) [25]. The present study also suggests that much of the adverse effect of a higher BMI on cardiovascular mortality could be ameliorated by blood pressure control, even in the absence of availability of other cardioprotective medications, such as statins.

Healthcare programs to increase the availability and use of blood pressure-lowering medication are being delivered in Cuba. However, the present study suggests that blood pressuring-lowering medication alone will not fully address the burden of cardiovascular mortality. Public health programs are also needed to address other major risk factors for cardiovascular disease, in particular smoking (about one-third of men and one-fifth of women smoke [26]), as well as the environmental determinants of health that are driving the increasing prevalence of overweight and obesity, and of diabetes, as reported in serial national surveys in Cuba [14].

## Conclusions

This large prospective study provides direct evidence for the effect of blood pressure, diabetes and BMI on cardiovascular mortality in Cuba. Overall, cardiovascular mortality doubled for each 20 mmHg higher usual SBP, or prior diabetes, or 10 kg/m^2^ higher usual BMI. These findings differ importantly from prospective studies conducted in other parts of Latin America, and highlight the need for more large prospective studies in the region. Despite comparatively low levels of blood pressure, diabetes and BMI in Cuba by international standards, the strength of their association with cardiovascular death means they nevertheless exert a substantial impact on premature mortality. As the levels of these risk factors increase in Cuba, so too will their importance as determinants of premature cardiovascular death, unless further efforts are made to address cardiovascular risk in this population.

## Supplementary Information

**Additional file 1.** Questionnaire (original Spanish), questionnaire (English translation), supplementary Tables S1-S5, and supplementary Figure S1-S11.

## Data Availability

The datasets generated and/or analysed during the current study are not publicly available, but are available from the corresponding author on reasonable request.

## Abbreviations

BMI: Body-mass index
SBP: Systolic blood pressure
DBP: Diastolic blood pressure
NCDs: Non-communicable diseases
ICD: International Classification of Disease
RR: Rate ratio
CI: Confidence interval

## References

[1] WHO Global action plan for the prevention and control of noncommunicable diseases 2013–2020. Geneva: World Health Organization; 2013.

[2] NCD Countdown 2030 Collaborators. NCD Countdown 2030: worldwide trends in non-communicable disease mortality and progress towards sustainable development goal target 3.4. Lancet. 2018;392(10152):1072–88. 10.1016/S0140-6736(18)31992-5. 10.1016/S0140-6736(18)31992-5 30264707

[3] Prospective Studies Collaboration. Age-specific relevance of usual blood pressure to vascular mortality: a meta-analysis of individual data for one million adults in 61 prospective studies. Lancet. 2002;360(9349):1903–13. 10.1016/S0140-6736(02)11911-8. 10.1016/s0140-6736(02)11911-8 12493255

[4] Prospective Studies Collaboration (2009). Body-mass index and cause-specific mortality in 900 000 adults: collaborative analyses of 57 prospective studies. Lancet.

[5] Global BMI Mortality Consortium (2016). Body-mass index and all-cause mortality: individual-participant-data meta-analysis of 239 prospective studies in four continents. Lancet.

[6] Asia Pacific Cohort Studies Collaboration. Sex-specific relevance of diabetes to occlusive vascular and other mortality: a collaborative meta-analysis of individual data from 980 793 adults from 68 prospective studies. Lancet Diabetes Endocrinol. 2018;6(7):538–46. 10.1016/S0140-6736(16)30175-1. 10.1016/S2213-8587(18)30079-2 PMC6008496 29752194

[7] Roth GA, Nguyen G, Forouzanfar MH, Mokdad AH, Naghavi M, Murray CJ (2015). Estimates of global and regional premature cardiovascular mortality in 2025. Circulation.

[8] Alegre-Diaz J, Herrington W, Lopez-Cervantes M (2016). Diabetes and cause-specific mortality in Mexico City. N Engl J Med.

[9] United Nations Population Fund. https://www.unfpa.org/data/world-population/CU. Accessed 1 Mar 2021

[10] Loewenberg S (2016). Cuba's focus on preventive medicine pays off. Lancet.

[11] Armas Rojas N, Lacey B, Lewington S, et al. Cohort Profile: the Cuba Prospective Study. Int J Epidemiol. 2019;48(3):680-1e. 10.1093/ije/dyy297. 10.1093/ije/dyy297 PMC6659378 30796445

[12] Clarke R, Shipley M, Lewington S, Youngman L, Collins R, Marmot M, Peto R (1999). Underestimation of risk associations due to regression dilution in long-term follow-up of prospective studies. Am J Epidemiol.

[13] Easton DF, Peto J, Babiker AG (1991). Floating absolute risk: an alternative to relative risk in survival and case-control analysis avoiding an arbitrary reference group. Stat Med.

[14] Mariano Bonet Gorbea M, Varona Pérez P. III Encuesta nacional de factores de riesgo y actividades preventivas de enfermedades no trasmisibles. Cuba 2010-2011. Havana: Editorial Ciencias Médicas; 2014.

[15] Rockhill B, Newman B, Weinberg C (1998). Use and misuse of population attributable fractions. Am J Public Health.

[16] Institute for Health Metrics and Evaluation. Global burden of disease (GBD). Global health data exchange. http://ghdx.healthdata.org/gbd-results-tool. Accessed 1 Mar 2021

[17] Emerging Risk Factors Collaboration (2010). Diabetes mellitus, fasting blood glucose concentration, and risk of vascular disease: a collaborative meta-analysis of 102 prospective studies. Lancet.

[18] Gajalakshmi V, Lacey B, Kanimozhi V, Sherliker P, Peto R, Lewington S (2018). Body-mass index, blood pressure, and cause-specific mortality in India: a prospective cohort study of 500 810 adults. Lancet Glob Health.

[19] Pischon T, Boeing H, Hoffmann K, Bergmann M, Schulze MB, Overvad K, van der Schouw YT, Spencer E, Moons KGM, Tjønneland A, Halkjaer J, Jensen MK, Stegger J, Clavel-Chapelon F, Boutron-Ruault MC, Chajes V, Linseisen J, Kaaks R, Trichopoulou A, Trichopoulos D, Bamia C, Sieri S, Palli D, Tumino R, Vineis P, Panico S, Peeters PHM, May AM, Bueno-de-Mesquita HB, van Duijnhoven FJB, Hallmans G, Weinehall L, Manjer J, Hedblad B, Lund E, Agudo A, Arriola L, Barricarte A, Navarro C, Martinez C, Quirós JR, Key T, Bingham S, Khaw KT, Boffetta P, Jenab M, Ferrari P, Riboli E (2008). General and abdominal adiposity and risk of death in Europe. N Engl J Med.

[20] Cameron AJ, Romaniuk H, Orellana L, Dallongeville J, Dobson AJ, Drygas W, Ferrario M, Ferrieres J, Giampaoli S, Gianfagna F, Iacoviello L, Jousilahti P, Kee F, Moitry M, Niiranen TJ, Pająk A, Palmieri L, Palosaari T, Satu M, Tamosiunas A, Thorand B, Toft U, Vanuzzo D, Veikko S, Veronesi G, Wilsgaard T, Kuulasmaa K, Söderberg S (2020). Combined influence of waist and hip circumference on risk of death in a large cohort of European and Australian adults. J Am Heart Assoc.

[21] Lacey B, Lewington S, Clarke R, Kong XL, Chen Y, Guo Y, Yang L, Bennett D, Bragg F, Bian Z, Wang S, Zhang H, Chen J, Walters RG, Collins R, Peto R, Li L, Chen Z, Chen J, Chen Z, Clarke R, Collins R, Guo Y, Li L, Lv J, Peto R, Walters R, Avery D, Bennett D, Boxall R, Bragg F, Chang Y, Chen Y, Chen Z, Clarke R, du H, Gilbert S, Hacker A, Holmes M, Iona A, Kartsonaki C, Kerosi R, Kurmi O, Lewington S, Lancaster G, Lin K, McDonnell J, Millwood I, Nie Q, Radhakrishnan J, Ryder P, Sansome S, Schmidt D, Sherliker P, Sohoni R, Stevens B, Turnbull I, Walters R, Wang J, Wang L, Wright N, Yang L, Yang X, Bian Z, Chen G, Guo Y, Han X, Hou C, Lv J, Pei P, Qu S, Tan Y, Yu C, Pang Z, Gao R, Wang S, Liu Y, du R, Zang Y, Cheng L, Tian X, Zhang H, Lv S, Wang J, Hou W, Yin J, Jiang G, Zhou X, Yang L, He H, Yu B, Li Y, Mu H, Xu Q, Dou M, Ren J, Wang S, Hu X, Wang H, Chen J, Fu Y, Fu Z, Wang X, Weng M, Zheng X, Li Y, Li H, Wang Y, Wu M, Zhou J, Tao R, Yang J, Ni C, Zhang J, Hu Y, Lu Y, Ma L, Tang A, Zhang S, Jin J, Liu J, Tang Z, Chen N, Huang Y, Li M, Meng J, Pan R, Jiang Q, Zhang W, Liu Y, Wei L, Zhou L, Chen N, Guan H, Wu X, Zhang N, Chen X, Tang X, Luo G, Li J, Chen X, Zhong X, Liu J, Sun Q, Ge P, Ren X, Dong C, Zhang H, Mao E, Wang X, Wang T, Zhang X, Zhang D, Zhou G, Feng S, Chang L, Fan L, Gao Y, He T, Sun H, He P, Hu C, Lv Q, Zhang X, Yu M, Hu R, Wang H, Qian Y, Wang C, Xie K, Chen L, Zhang Y, Pan D, Huang Y, Chen B, Yin L, Jin D, Liu H, Fu Z, Xu Q, Xu X, Zhang H, Xiong Y, Long H, Li X, Zhang L, Qiu Z (2018). Age-specific association between blood pressure and vascular and non-vascular chronic diseases in 0.5 million adults in China: a prospective cohort study. Lancet Glob Health.

[22] Chen Z, Iona A, Parish S, Chen Y, Guo Y, Bragg F, Yang L, Bian Z, Holmes MV, Lewington S, Lacey B, Gao R, Liu F, Zhang Z, Chen J, Walters RG, Collins R, Clarke R, Peto R, Li L, Chen J, Chen Z, Clarke R, Collins R, Guo Y, Li L, Lv J, Peto R, Walters R, Avery D, Bennett D, Boxall R, Bragg F, Chang Y, Chen Y, Chen Z, Clarke R, du H, Gilbert S, Hacker A, Holmes M, Iona A, Kartsonaki C, Kerosi R, Kurmi O, Lewington S, Lancaster G, Lin K, McDonnell J, Millwood I, Nie Q, Radhakrishnan J, Ryder P, Sansome S, Schmidt D, Sherliker P, Sohoni R, Stevens B, Turnbull I, Walters R, Wang J, Wang L, Wright N, Yang L, Yang X, Bian Z, Chen G, Guo Y, Han X, Hou C, Lv J, Pei P, Qu S, Tan Y, Yu C, Pang Z, Gao R, Wang S, Liu Y, du R, Zang Y, Cheng L, Tian X, Zhang H, Lv S, Wang J, Hou W, Yin J, Jiang G, Zhou X, Yang L, He H, Yu B, Li Y, Mu H, Xu Q, Dou M, Ren J, Wang S, Hu X, Wang H, Chen J, Fu Y, Fu Z, Wang X, Weng M, Zheng X, Li Y, Li H, Wang Y, Wu M, Zhou J, Tao R, Yang J, Ni C, Zhang J, Hu Y, Lu Y, Ma L, Tang A, Zhang S, Jin J, Liu J, Tang Z, Chen N, Huang Y, Li M, Meng J, Pan R, Jiang Q, Zhang W, Liu Y, Wei L, Zhou L, Chen N, Guan H, Wu X, Zhang N, Chen X, Tang X, Luo G, Li J, Chen X, Zhong X, Liu J, Sun Q, Ge P, Ren X, Dong C, Zhang H, Mao E, Wang X, Wang T, Zhang X, Zhang D, Zhou G, Feng S, Chang L, Fan L, Gao Y, He T, Sun H, He P, Hu C, Lv Q, Zhang X, Yu M, Hu R, Wang H, Qian Y, Wang C, Xie K, Chen L, Zhang Y, Pan D, Huang Y, Chen B, Yin L, Jin D, Liu H, Fu Z, Xu Q, Xu X, Zhang H, Xiong Y, Long H, Li X, Zhang L, Qiu Z (2018). Adiposity and risk of ischaemic and haemorrhagic stroke in 0.5 million Chinese men and women: a prospective cohort study. Lancet Glob Health.

[23] Kroll ME, Green J, Beral V, Sudlow CLM, Brown A, Kirichek O, Price A, Yang TYO, Reeves GK, For the Million Women Study Collaborators (2016). Adiposity and ischemic and hemorrhagic stroke: prospective study in women and meta-analysis. Neurology.

[24] Law MR, Morris JK, Wald NJ (2009). Use of blood pressure lowering drugs in the prevention of cardiovascular disease: meta-analysis of 147 randomised trials in the context of expectations from prospective epidemiological studies. BMJ.

[25] Armas Rojas N, Dobell E, Lacey B, Varona-Pérez P, Burrett JA, Lorenzo-Vázquez E, Calderón Martínez M, Sherliker P, Bess Constantén S, Morales Rigau JM, Hernández López OJ, Martínez Morales MÁ, Alonso Alomá I, Achiong Estupiñan F, Díaz González M, Rosquete Muñoz N, Cendra Asencio M, Peto R, Emberson J, Dueñas Herrera A, Lewington S (2019). Burden of hypertension and associated risks for cardiovascular mortality in Cuba: a prospective cohort study. Lancet Public Health.

[26] World Bank. https://data.worldbank.org/indicator/SH.PRV.SMOK.FE?locations=CU. Accessed 1 Mar 2021

