# Supplementary material for: Body-mass index, blood pressure, diabetes and cardiovascular mortality in Cuba: prospective study of 146,556 participants
Source: BMC Public Health. 2021 May 27;21:963. doi: 10.1186/s12889-021-10911-9 (PMC8157418; doi:10.1186/s12889-021-10911-9)
Supplement: Supplementary file 1 — Additional file 1. Questionnaire (original Spanish), questionnaire (English translation), supplementary Tables S1-S5, and supplementary Figure S1-S11. [file 12889_2021_10911_MOESM1_ESM.pdf]

Blood pressure, diabetes, body-mass index, and cardiovascular mortality in Cuba: prospective study of 146 556 adults

Supplementary Appendix, Table of Contents

	Page
**Questionnaire** (Spanish)	2
**Questionnaire** (English translation)	3
**Table S1.** Mortality endpoints and ICD codes	4
**Table S2.** Number of participants exlcuded from mortality analyses, by sex	5
**Table S3.** Blood pressure, diabetes and BMI at baseline, by age and sex	6
**Table S4.** Baseline characteristics, by BMI (7 categories)	7
**Table S5.** Blood pressure, diabetes and BMI of resurveyed participants, by age at baseline and at resurvey	8
**Figure S1.** Baseline vs resurvey values of systolic blood pressure (SBP), diastolic blood pressure (DBP) and BMI, in groups defined by baseline quintiles of each measure, among resurveyed participants	9
**Table S6.** Estimated long-term average ('usual') values of systolic blood pressure (SBP), diastolic blood pressure (DBP) and BMI	10
**Figure S2.** Vascular mortality vs usual systolic blood pressure (SBP), usual diastolic blood pressure (DBP), and usual BMI at ages 35-79 years in Cuba	11
**Figure S3.** Ischaemic heart disease and stroke mortality vs usual systolic blood pressure (SBP) at ages 35-79 years in Cuba	12
**Figure S4.** Ischaemic heart disease and stroke mortality vs usual BMI at ages 35-79 years in Cuba	13
**Figure S5.** Death rate ratios (RR) for vascular mortality vs usual systolic blood pressure (SBP), diabetes and usual BMI, at ages 35-79 years in Cuba, with further exclusion of those with non-vascular chronic disease at baseline	14
**Figure S6.** Vascular mortality vs usual systolic blood pressure (SBP) and usual BMI, at ages 35-79 years in Cuba, with further exclusion of those with non-vascular chronic disease at baseline	15
**Figure S7.** Death rate ratios (RR) for vascular mortality vs usual systolic blood pressure (SBP), diabetes and usual BMI, at ages 35-79 years in Cuba, not excluding any follow-up	16
**Figure S8.** Vascular mortality vs usual systolic blood pressure (SBP) and usual BMI, at ages 35-79 years in Cuba, not excluding any follow-up	17
**Figure S9.** Death rate ratios (RR) for vascular mortality vs usual systolic blood pressure (SBP), diabetes and usual BMI, at ages 35-79 years in Cuba, excluding those taking blood pressure-lowering medication at baseline	18
**Figure S10.** Vascular mortality vs usual systolic blood pressure (SBP) and usual BMI, at ages 35-79 years in Cuba, excluding those taking blood pressure-lowering medication at baseline	19
**Figure S11.** Death rate ratios (RR) for vascular mortality (excluding ischaemic heart disease and stroke) vs usual systolic blood pressure (SBP), diabetes and usual BMI at ages 35-79 years in Cuba	20

ESTUDIO CUBANO SOBRE TABACO Y SALUD

Provincia No. ☐ 1-9 Municipio No. ☐☐ 0-99 Area de Salud No. ☐ 0-9

№ 17524 **A**

Carnet de Identidad No. |_|_|_|_|_|_|_|–|_|_|_|_|_|
Fecha de nacimiento (a.m.d) + 5 números
(11 números: excluir si no tiene C.I. de 6 + 5 números)

Nombre de la persona y dirección: |_|_|_|_|_|_|_|_|_|_|_|_|_|_|_|_|_|_|_|_|_|_|_|_|_|_|_|_|_|_|

Calle No. Teléfono

Nombre del familiar y dirección: |_|_|_|_|_|_|_|_|_|_|_|_|_|_|_|_|_|_|_|_|_|_|_|_|_|_|_|_|_|_|

Calle No. Municipio Provincia Teléfono

LETRA DE MOLDE, POR FAVOR

Sexo (1 = masc, 2 = fem.) ☐ 1 - 2

Color de piel (1 = blanco, 2 = negro, 3 = mestizo, 4 = otra) ☐ 1 - 4

Estado social (1 = soltero/viudo, 2 = casado) ☐ 1 - 2

Educación completada (0 = ninguno*, 1 = primaria, 2 = secundaria, 3 = técnico medio, 4 = obrero calificado, 5 = pre-universitario, 6 = universitario) ☐ 0 - 6

Categoría ocupacional (0 = ninguno*, 1 = dirigente, 2 = profesional, 3 = técnico, 4 = de servicio, 5 = agrícola, 6 = industria, 7 = militar) ☐ 0 - 7

* Si es cero (Ej. ama de casa, jubilado o desempleado), diga si ha trabajado alguna vez. ☐ 0 - 7

Quisiera medir su presión arterial mientras esta sentado, hacerle unas pocas preguntas sobre tabaco y alcohol, y medir su PA de nuevo.

Presión arterial (una sola toma, sentado): |_|_|_| / |_|_|_| PAS / PAD

Ahora, TABACO: ¿Ha fumado alguna vez regularmente (Ej. en la mayoría de los días)?
SI NO, vaya directamente a las preguntas sobre ALCOHOL. ☐ S=Sí, N=No

¿A QUE EDAD APROXIMADAMENTE COMENZO USTED A FUMAR REGULARMENTE? ☐☐ años de edad

¿Desde entonces ha dejado de fumar alguna vez por un año o más? ☐ S=Sí, N=No

¿Aproximadamente cuántos cigarrillos por día se fumó usted el último mes? (0 = ninguno) ☐☐ diariamente

¿Aproximadamente cuántos tabacos por día se fumó usted el último mes? (0 = ninguno) ☐☐ diariamente

¿Si no fumó el último mes, a que edad dejó usted de fumar? (en blanco si no es aplicable) ☐☐ años de edad

Ahora me gustaría hacerle algunas preguntas sobre ALCOHOL:

¿Aproximadamente cuántos días a la semana toma usted ron u otra bebida fuerte? (0 = cero días o usualmente menos de una vez por semana) ☐ 0 - 7

¿El día que usted toma ron (u otra bebida fuerte) cuántas líneas se toma? ☐☐ líneas

¿Aproximadamente cuántos días a la semana toma usted cerveza? (0 = cero días o usualmente menos de una vez por semana) ☐ 0 - 7

¿El día que usted toma cerveza, cuántas botellas se toma (aprox.) ☐☐ botellas

Finalmente, algunos aspectos médicos: Quisiera preguntarle sobre algunas enfermedades. Lo que quiero conocer es si usted alguna vez ha sido tratado por alguna de estas enfermedades. Si no lo ha sido, conteste No (N).

Asma	☐ S/N	A.V.E. (definitivo/probable)	☐ S/N	Cirrosis	☐ S/N
Enfisema (E.P.O.C.)	☐ S/N	Infarto miocardio "	☐ S/N	Enf. renal crónica	☐ S/N
Diabetes	☐ S/N	Angina pectoris "	☐ S/N	Cáncer de piel	☐ S/N
Ulcera péptica	☐ S/N	Hipertensión arterial	☐ S/N	Otro cáncer*	☐ S/N

¿*Si Otro Cáncer = Sí, dónde le comenzó el cáncer? ☐ 0 - 9

(1 = boca o faringe, 2 = laringe, 3 = pulmón, 4 = esófago, 5 = estómago, 6 = páncreas, 7 = cuello útero, 8 = vejiga, 9 = mama, 0 = otro, más de un sitio, o no conocido)

¿Esta usted tomando alguna medicina regularmente desde hace más de un mes? Si es Sí: ¿Qué medicinas esta tomando? (Marque con X)

Digoxina	☐	Nitratos	☐	Hipoglic. oral	☐	Anticoncept.	☐	Ca. Antag.	☐
Beta B.	☐	Inh. ACE	☐	Insulina	☐	Antidepres.	☐	Broncodilat.	☐
Diurético	☐	ASA	☐	Esteroides	☐	Ansiolítico	☐	Antiácido/H2	☐

MUCHAS GRACÍAS POR SUS RESPUESTAS: Ahora necesito tomarle de nuevo su presión arterial. Repita la toma de PA (una sola toma sentado): |_|_|_| / |_|_|_| PAS/PAD

¿Cuál es la última cifra PA que tiene en su historia clínica antes de hoy?

Fecha de la última PA (a.m.d) |_|_|_|_|_|_| & |_|_|_| / |_|_|_| PAS/PAD

¿Cuál es su *TALLA* aproximada? (medirlo o preguntarlo) |_|_|_| cm

¿Cuál es su *PESO* aproximado? (pesarlo o preguntarlo) |_|_|_| kg

Fecha de la entrevista |_|_|_|_|_|_| (a.m.d)

Por favor chequee TODA la encuesta ¡MUCHAS GRACIAS!

Calidad de la cooperación (1 = pobre, 2 = regular, 3 = buena) ☐ 1 - 3

Nombre y apellidos del entrevistador: |_|_|_|_|_|_|_|_|_|_|_|_|_|_|_|_|_|_|_|

¿Algún comentario? ....

PARA LA SIGUIENTE PERSONA, UTILICE EL REVERSO DE ESTA PAGINA →

CUBAN STUDY OF TOBACCO AND HEALTH: PROSPECTIVE

Province № ☐ 1-9 Municipality № ☐☐ 0-99 Health Area № ☐ 0-9 Identity Card № ☐☐☐☐☐☐-☐☐☐☐☐ Date of birth (y,m,d)

Name of interviewee: ________________________________

Address: ________________________________
Street № Municipality Province Telephone

Name of friend: ________________________________

Address: ________________________________
Street № Municipality Province Telephone

CAPITAL LETTERS, PLEASE!

Sex (1=masc, 2=fem) ☐ 1-2

Skin colour(1=white, 2=black, 3=mixed, 4=other) ☐ 1-4

Marital status (1=single/widowed, 2=married) ☐ 1-2

Education completed (0=none, 1=primary, 2=secondary, 3=technician, 4=skilled worker, 5=high school, 6=university) ☐ 0-6

Occupational category (0=none, 1=manager, 2=professional, 3=technician, 4=service worker, 5=agricultural, 6=industrial, 7=military; if housewife, retired or unemployed, we would like to know if you were employed in the past) ☐ 0-7

(☐ 0-7)

What I'd like to do is measure your blood pressure while you are sitting down, ask you a few questions about tobacco and alcohol, and then do your blood pressure again. Blood pressure (single measurement, seated): ☐☐☐ / ☐☐☐ SBP / DBP

Now, TOBACCO: Have you EVER smoked regularly (i.e. on most days for at least a year)? If NO, then go directly to the ALCOHOL questions ☐ **Y=Yes, N=No**

AT ABOUT WHAT AGE DID YOU FIRST START SMOKING TOBACCO REGULARLY? ☐☐ years of age

Since then, have you ever STOPPED smoking for a year or more? ☐ Y=Yes, N=No

About how many cigarettes a day did you smoke last month? (0=none) ☐☐ daily

About how many cigars a day did you smoke last month? (0=none) ☐☐ daily

If NO SMOKING last month, at about what age did you last stop smoking? (blank = not applicable) ☐☐ years of age

Now I'd like to ask you some questions about ALCOHOL:

About how many days a week would you drink any rum or other spirits? (0=none or usually less than once a week) ☐ 0-7

On a day when you drink rum (or other spirits), about how many glasses ("lineas") would you drink? ☐☐ lineas

About how many days a week would you drink beer? (0=none or usually less than once a week) ☐ 0-7

On a day when you do drink beer, about how many bottles would you drink? ☐☐ bottles

Finally, MEDICAL MATTERS: I'd like to ask about various different illnesses. What I want to know for each illness is whether you've ever had medical attention for it. If you've never heard of the illness, then just say NO.

Asthma	☐ Y/N	Stroke (definite/probable)	☐ Y/N	Cirrhosis of the liver	☐ Y/N
Emphysema/C.O.P.D.	☐ Y/N	Myocardial infarction "	☐ Y/N	Chronic kidney disease	☐ Y/N
Diabetes	☐ Y/N	Angina pectoris "	☐ Y/N	Skin cancer	☐ Y/N
Peptic ulcer	☐ Y/N	Hypertension	☐ Y/N	Other cancer*	☐ Y/N

*If Other cancer, where in your body did the cancer start? ☐ 0-9
(1=mouth, 2=larynx, 3=lung, 4=oesphagus, 5=stomach, 6=pancreas, 7=cervix, 8=bladder, 9=breast, 0=other, more than 1 site, or not known)

Have you been taking any regular medication for more than 1 month? If Yes: What such medicines are you now taking? (Mark with an **X**)

Digoxin ☐	Nitrates ☐	Oral hypoglycaemic ☐	O.Contraceptive ☐	Ca. antagonist ☐
Beta blocker ☐	ACE inhibitor ☐	Insulin ☐	Antidepressant ☐	Bronchodilator ☐
Diuretic ☐	Aspirin ☐	Steroids ☐	Tranquillisers ☐	Antacid/H2 ☐

THANK YOU VERY MUCH FOR YOUR ANSWERS. Now we just need one more blood pressure. Repeat blood pressure (single measurement, seated) ☐☐☐ / ☐☐☐ PAS / PAD

What was the last blood pressure in the patient's medical record **before** today?

Date of last BP (y,m,d) ☐☐☐☐☐☐ & ☐☐☐ / ☐☐☐ PAS / PAD

What is your approximate **HEIGHT** (either ask or measure)? ☐☐☐ cm

What is your approximate **WEIGHT** (either ask or measure)? ☐☐☐ kg

Date of interview ☐☐☐☐☐☐ (y,m,d)

Quality of co-operation (1=poor, 2=fair, 3=good) ☐ 1-3

Please check that **ALL** the questions have been answered, so that there is no need to write back to you for extra details.
MANY THANKS!

Name of interviewer: please PRINT ________________________________

Any comments: ....

Table S1. Mortality endpoints and ICD codes

Endpoint name	ICD-9 codes	ICD-10 codes
Ischaemic heart disease	410-414	I20-I25
Stroke	430-431, 433-434, 436	I60-I61, I63-I64
Other vascular	390-409, 415-429, 432, 435, 437-459, 798	I00-I19, I26-I59, I62, I65-I99, R96
Rheumatic heart disease	390-398	I00-I09
Hypertensive disease	401-405	I10-I15
Pulmonary embolism	415, 450	I26
Inflammatory heart disease	420-424	I30-I33, I38-I43
Cardiac arrhythmia	426-427	I44-I45, I47-I49
Heart failure	428	I50
Other cerebrovascular (not stroke)	432, 435, 437-438	I62, I65-I69
Atherosclerosis	440, 442-448	I70, I72-I79
Aortic aneurysm	441	I71
Other	399-400, 406-409, 416-419, 425, 429, 439, 449, 451-459, 798	I16-I19, I27-I29, I34-I37, I46, I51-I59, I80-I99, R96
All vascular causes	390-459, 798	I00-I99, R96

Table S2. Number of participants excluded from mortality analyses, by sex

	Men	Women	All
**Number of participants recruited**	64 743	81 813	146 556
**Reason for exclusion:**			
**Prior vascular disease***	4331	5722	10 053
**Systolic blood pressure**			
Missing	122	170	292
<80 mm Hg	12	49	61
≥250 mmHg	2	4	6
**Diastolic blood pressure**			
Missing	167	227	394
<40 mm Hg	3	8	11
≥150 mmHg	3	7	10
**BMI**			
Missing	619	2178	2797
<15 kg/m^2	217	431	648
≥40 kg/m2	275	624	899
**Diabetic status**			
Missing	0	0	0
**Missing values in covariates**			
Age at baseline	0	0	0
Sex	0	0	0
Province	0	0	0
Level of education	61	103	164
Smoking status	0	0	0
Alcohol status	0	0	0
**No follow-up at ages 35-79 years**			
Age 80 years or over at baseline	3333	4156	7489
Lost to follow-up at baseline†	116	116	232
**Total participants excluded**	8346	12 271	20 617
**Total participants in main analyses**	**56 397**	**69 542**	**125 939**

*At baseline screening, history of any of myocardial infarction, angina, stroke, chronic obstructive pulmonary disease, cirrhosis of the liver, chronic kidney disease, any cancer (including skin), peptic ulcer.

†Uncertainty with identification number given at baseline, which was required to identify death.

Table S3. Blood pressure, diabetes, and BMI at baseline, by age and sex

Age at baseline, years	Mean age, years	Mean SBP, mmHg	Mean DBP, mmHg	Diabetes prevalence, %	Mean BMI, kg/m^2
**Men**					
35-39	37	122	79	1.6	23.9
40-44	43	123	80	1.9	23.9
45-49	47	124	81	2.5	24.1
50-54	52	125	81	3.4	24.2
55-59	57	126	82	4.2	24.2
60-64	62	127	82	5.3	24.1
65-69	67	128	81	6.5	23.9
70-74	72	128	81	6.7	23.8
75-79	77	128	81	7.3	23.6
**Women**					
35-39	37	117	76	2.1	23.9
40-44	43	119	77	3.0	24.1
45-49	47	122	79	4.0	24.3
50-54	52	124	80	6.0	24.6
55-59	57	127	81	8.1	24.7
60-64	62	128	82	10.6	24.7
65-69	67	130	82	13.0	24.5
70-74	72	130	81	13.1	24.2
75-79	77	130	81	13.6	23.9

SBP=systolic blood pressure; DBP=diastolic blood pressure. Exclusions as in Table 1.

Table S4. Baseline characteristics, by BMI (7 categories)

	Baseline BMI, kg/m^2							Overall
	**15-<20**	**20-<22.5**	**22.5-<25**	**25-<27.5**	**27.5-<30**	**30-<35**	**35-<40**	
Number of participants	11 846	29 537	40 959	24 123	11 375	6747	1352	125 939
Female	61%	54%	51%	54%	59%	67%	74%	55%
Age, years	53 (13)	52 (12)	52 (12)	52 (12)	53 (12)	54 (12)	54 (11)	53 (12)
BMI, kg/m^2	18.6 (1.2)	21.4 (0.7)	23.7 (0.7)	26.1 (0.7)	28.5 (0.7)	31.8 (1.3)	36.9 (1.4)	24.2 (3.6)
Height, cm	166 (9)	165 (9)	164 (9)	164 (9)	163 (10)	160 (10)	154 (12)	164 (9)
SBP, mmHg	121 (15)	122 (14)	124 (14)	126 (15)	128 (17)	129 (18)	131 (18)	124 (15)
Diabetes	4%	4%	5%	6%	7%	8%	10%	5%
No formal education beyond primary school	36%	34%	31%	31%	32%	34%	32%	33%
Current smoker (in men)	55%	50%	46%	42%	38%	34%	31%	46%
Current smoker (in women)	37%	30%	26%	24%	22%	22%	22%	27%
Alcohol intake at least weekly	16%	16%	16%	16%	15%	15%	13%	16%
Taking anti-hypertensives	11%	12%	15%	19%	22%	26%	29%	16%

Data are n (%) or mean (SD). Results are standardised to the age and sex of the 125 939 participants. Participants with no follow-up at ages 35-79 years, those with pre-existing vascular disease at baseline, and those with incomplete information on SBP, diabetes, BMI or covariates were excluded.

Table S5. Blood pressure, diabetes and BMI of resurveyed participants, by age at baseline and at resurvey

A. Baseline blood pressure, diabetes, and BMI, by age at baseline

Age at baseline, years	Participants	Mean age, years	Mean SBP, mmHg	Mean DBP, mmHg	Diabetes prevalence, %	Mean BMI, kg/m^2
40-44	524	42	119	78	2.5	24.7
45-49	564	48	122	80	2.3	24.9
50-54	573	52	127	82	4.5	25.2
55-59	454	58	129	83	6.4	25.2
60-64	362	62	130	82	7.2	25.0
65-69	228	67	133	84	8.8	25.3
70-74	158	72	135	83	8.9	24.8
75-79	136	77	135	83	8.8	24.4
All	2999	55	126	81	5.1	25.0

B. Resurvey blood pressure, diabetes, and BMI, by age at resurvey

Age at resurvey, years	Participants	Mean age, years	Mean SBP, mmHg	Mean DBP, mmHg	Diabetes prevalence, %	Mean BMI, kg/m^2
40-44	477	43	117	77	2.9	25.5
45-49	556	47	120	78	3.6	25.5
50-54	544	53	124	80	7.9	25.5
55-59	569	57	127	81	8.4	25.5
60-64	493	62	127	80	14.8	25.8
65-69	390	67	131	81	11.8	25.3
70-74	264	72	130	80	16.7	25.2
75-79	191	77	132	79	13.6	25.0
All*	3484	55	124	79	8.0	25.5

SBP=systolic blood pressure; DBP=diastolic blood pressure. Analyses at baseline and at resurvey were restricted to participants ages 40-79 as there were few participants aged less than 40 at resurvey. Exclusions as in Table 1. *Standardised to the baseline age distribution of resurvey participants

Figure S1. Baseline versus resurvey values of systolic blood pressure (SBP), diastolic blood pressure (DBP), and BMI, in groups defined by baseline quintiles of each measure, among resurveyed participants

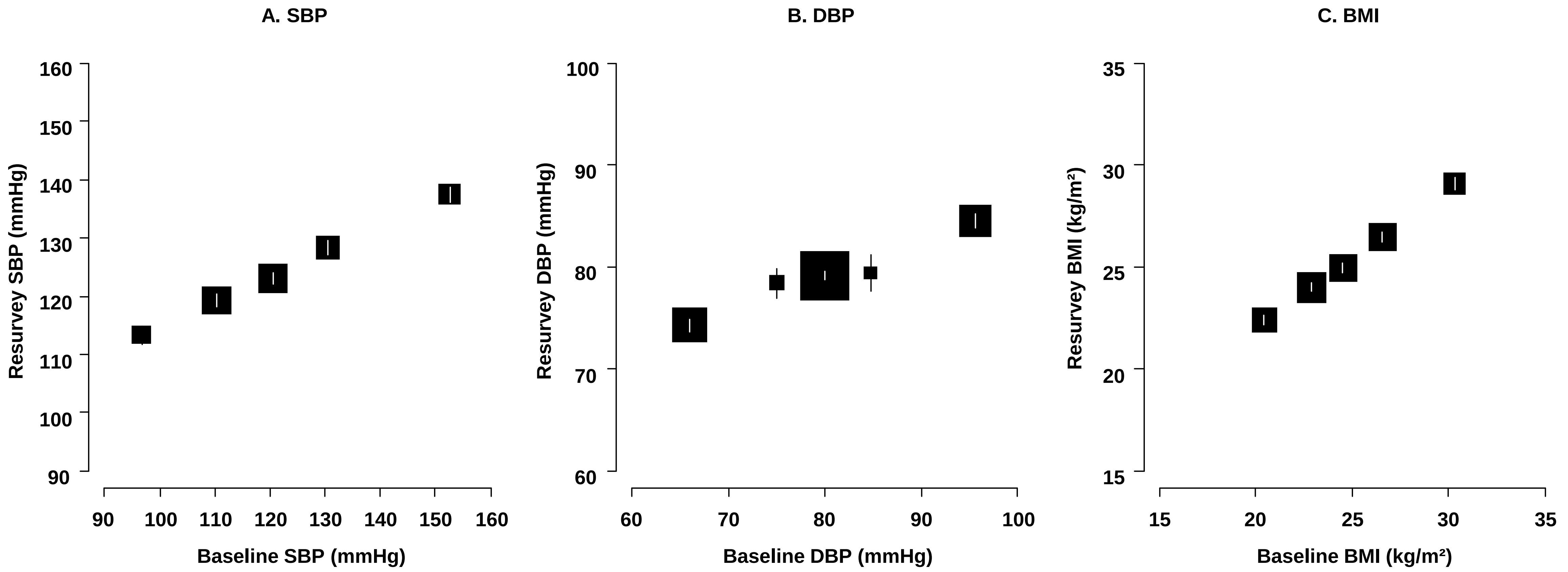

Exclusions as in Table 1.

Table S6. Estimated long-term average ('usual') values of systolic blood pressure (SBP), diastolic blood pressure (DBP) and BMI

A. SBP

Baseline SBP categories, mmHg	Mean SBP at baseline, mmHg	Estimated usual SBP, mmHg
80-<125	114.9	119.7
125-<145	132.9	128.3
145-<165	153.0	138.0
165-<250	178.3	150.1
All	124.1	124.1

B. DBP

Baseline DBP categories, mmHg	Mean DBP at baseline, mmHg	Estimated usual DBP, mmHg
40-<75	66.9	74.1
75-<85	79.6	79.7
85-<95	88.9	83.9
95-<150	101.5	89.4
All	79.8	79.8

C. BMI

Baseline BMI categories, kg/m^2	Mean BMI at baseline, kg/m^2	Estimated usual BMI, kg/m^2
15-<20	18.6	20.8
20-<22.5	21.4	22.6
22.5-<25	23.7	23.9
25<27.5	26.1	25.3
27.5-<30	28.5	26.7
30-<35	31.8	28.7
35-<40	36.8	31.7
All	24.2	24.2

Exclusions as in Table 1. Usual SBP, usual DBP and usual BMI were estimated by correcting mean SBP, mean DBP and mean BMI at baseline using regression dilution ratios of 0.48, 0.44 and 0.59 respectively.

Figure S2. Vascular mortality vs usual systolic blood pressure (SBP), usual diastolic blood pressure (DBP), and usual BMI at ages 35-79 years in Cuba

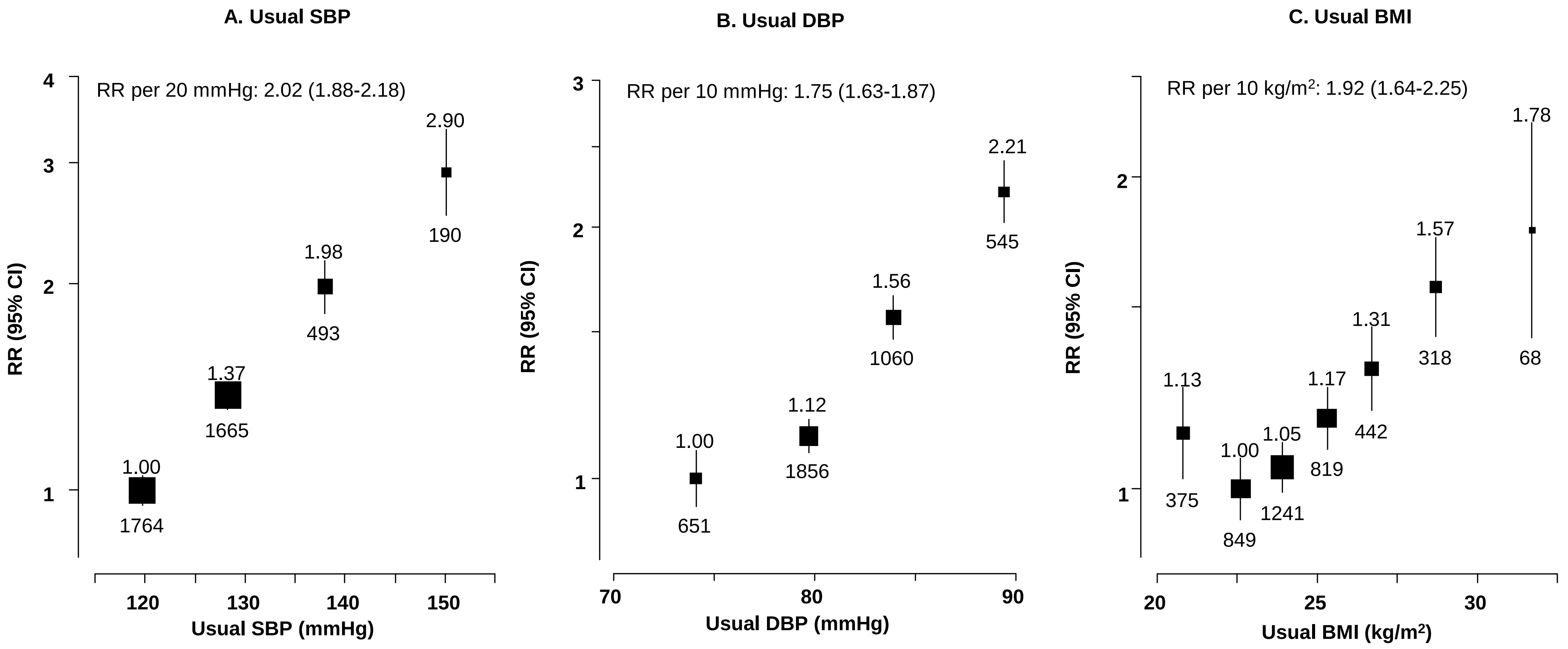

Rate ratios (RR) adjusted for age, sex, education, province, smoking, alcohol, and BMI (when appropriate), and omit the first 5 years of follow up. Exclusions as in Table 1.
For each category, area of the square is inversely proportional to the variance of the category−specific log risk, which also determines the confidence interval (CI).

Figure S3. Ischaemic heart disease and stroke mortality vs usual systolic blood pressure (SBP) at ages 35-79 years in Cuba

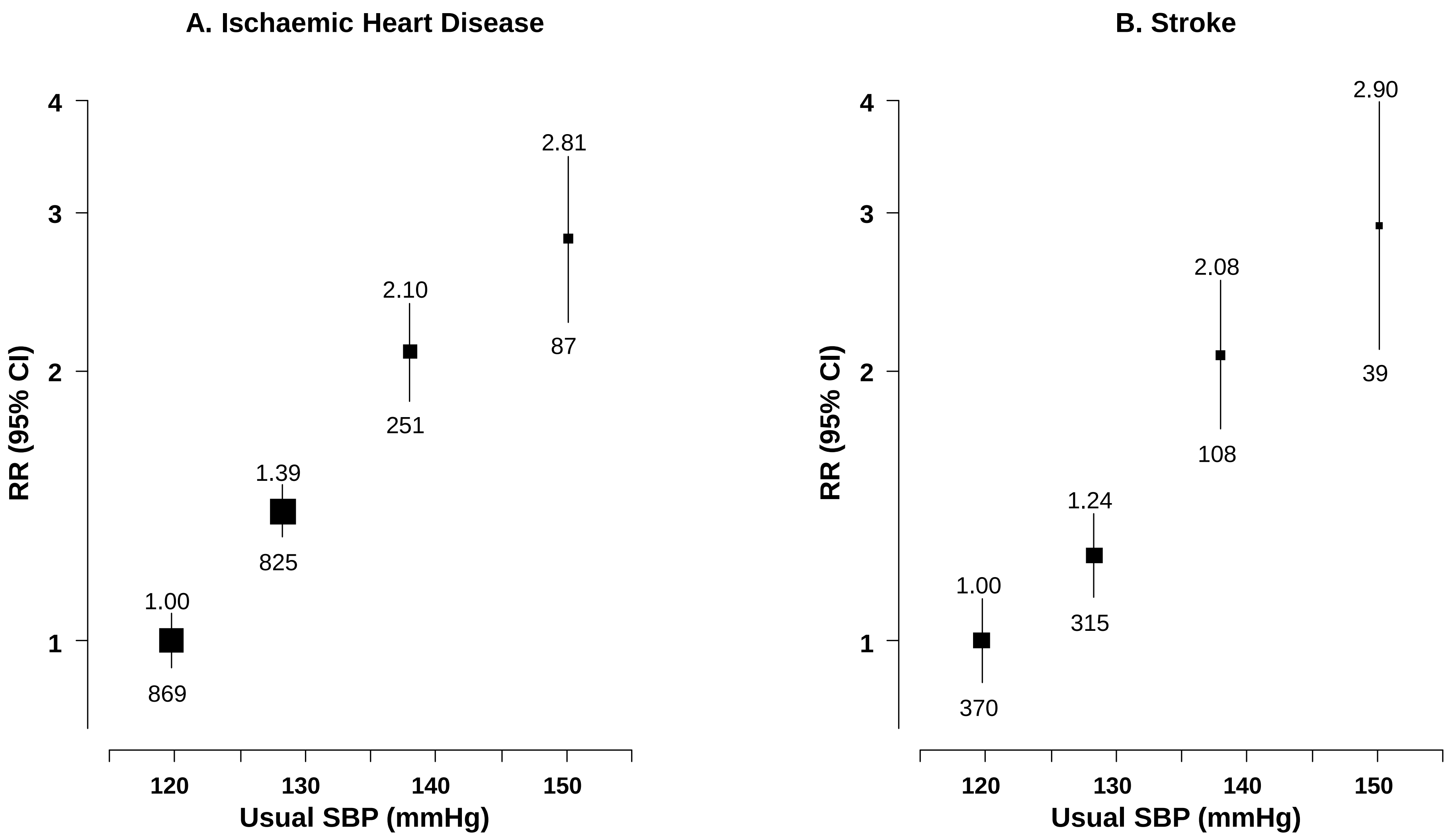

Rate ratios (RR) adjusted for age, sex, education, province, smoking, alcohol, and BMI, and omit the first 5 years of follow up. Exclusions as in Table 1. For each SBP category, area of the square is inversely proportional to the variance of the category-specific log risk, which also determines the confidence interval (CI).

Figure S4. Ischaemic heart disease and stroke mortality vs usual BMI at ages 35-79 years in Cuba

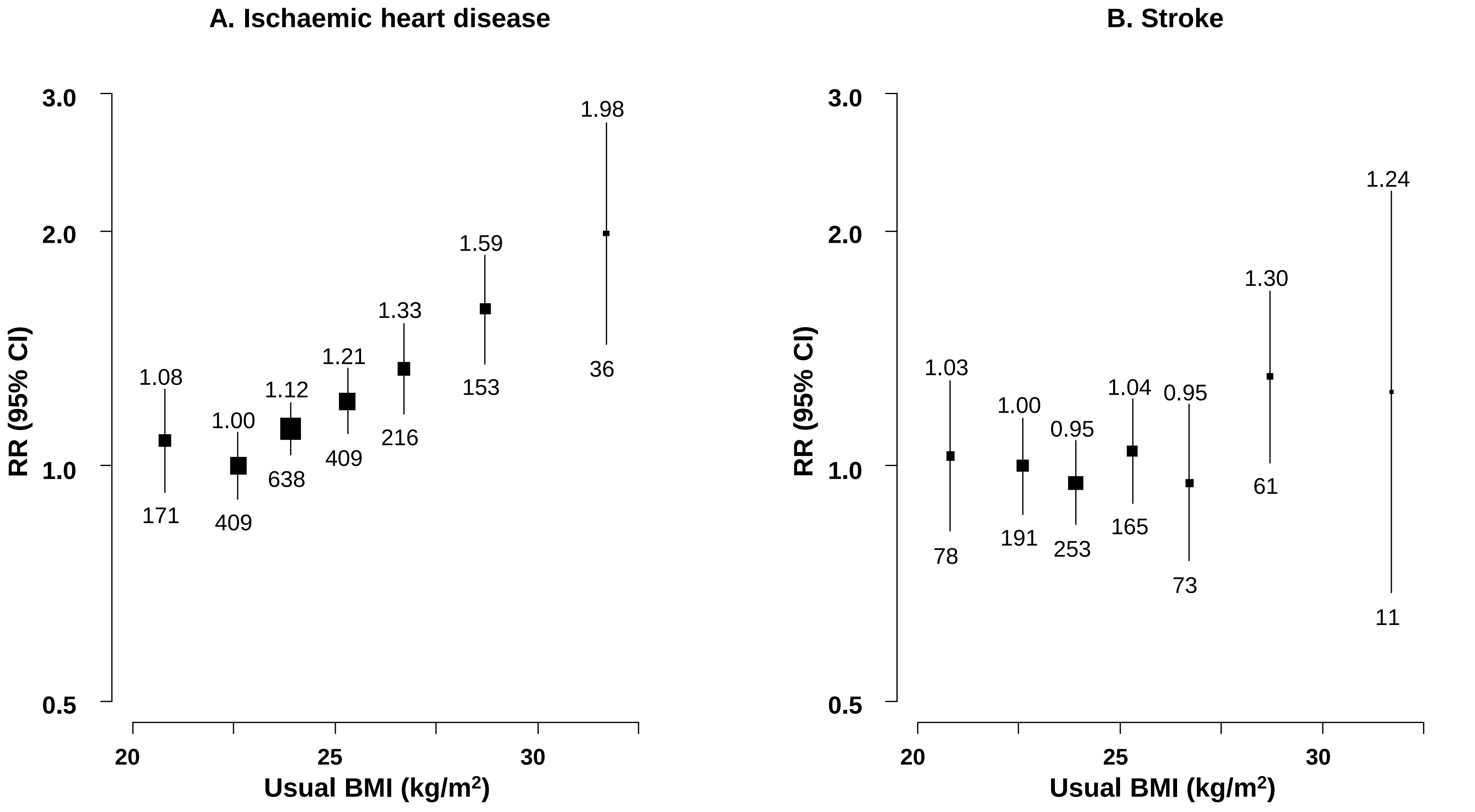

Rate ratios (RR) adjusted for age, sex, education, province, smoking, and alcohol, and omit the first 5 years of follow up. Exclusions as in Table 1. For each BMI category, area of the square is inversely proportional to the variance of the category-specific log risk, which also determines the confidence interval (CI).

Figure S5. Death rate ratios (RR) for vascular mortality vs usual systolic blood pressure (SBP), diabetes, and usual BMI, at ages 35-79 years in Cuba, with further exclusion of those with non-vascular chronic disease at baseline

	Mean age at death (years)	Deaths	RR (CI) per 20 mmHg higher usual SBP	RR (CI) for diabetes vs no diabetes	RR (CI) per 10 kg/m² higher usual BMI
**Age-at-risk (years)**					
35-59	55	564	3.54 (2.86 - 4.38)	2.42 (1.69 - 3.47)	1.93 (1.24 - 3.02)
60-69	66	1170	2.29 (1.99 - 2.63)	2.76 (2.29 - 3.33)	1.92 (1.43 - 2.59)
70-79	75	2046	1.71 (1.54 - 1.90)	1.94 (1.70 - 2.21)	1.89 (1.52 - 2.36)
			Trend test: χ^2_1=38.6 (p<0.001)	Trend test: χ^2_1=6.3 (p=0.01)	Trend test: χ^2_1=0.0 (p=0.9)
**Sex**					
Men	69	1956	2.10 (1.87 - 2.35)	2.12 (1.78 - 2.52)	1.93 (1.50 - 2.48)
Women	70	1824	1.97 (1.78 - 2.20)	2.21 (1.94 - 2.51)	1.86 (1.49 - 2.31)
			Heterogeneity test: χ^2_1=0.6 (p=0.4)	Heterogeneity test: χ^2_1=0.1 (p=0.7)	Heterogeneity test: χ^2_1=0.1 (p=0.8)
**Smoking**					
Never	70	1787	1.92 (1.72 - 2.15)	2.30 (2.01 - 2.63)	2.07 (1.65 - 2.59)
Ever	68	1993	2.14 (1.92 - 2.39)	2.02 (1.72 - 2.39)	1.72 (1.35 - 2.20)
			Heterogeneity test: χ^2_1=1.8 (p=0.2)	Heterogeneity test: χ^2_1=1.4 (p=0.2)	Heterogeneity test: χ^2_1=1.1 (p=0.3)
**Subtype**					
IHD	69	1871	2.09 (1.87 - 2.34)	2.36 (2.04 - 2.73)	1.85 (1.46 - 2.34)
Stroke	69	766	1.88 (1.57 - 2.24)	1.89 (1.49 - 2.41)	1.23 (0.84 - 1.81)
Other vascular	70	1143	2.05 (1.78 - 2.35)	2.10 (1.74 - 2.54)	2.65 (1.98 - 3.55)
**Overall**	**69**	**3780**	**2.03 (1.88 - 2.20)**	**2.18 (1.97 - 2.42)**	**1.91 (1.62 - 2.25)**

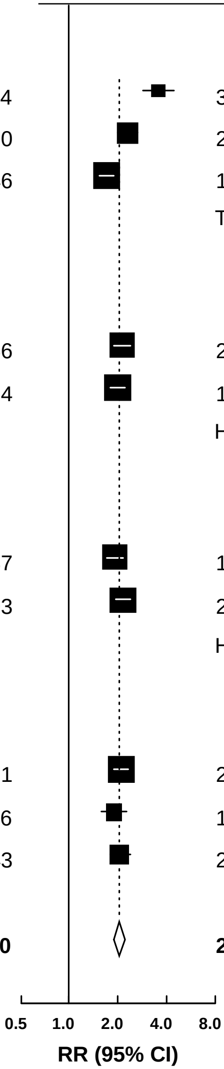

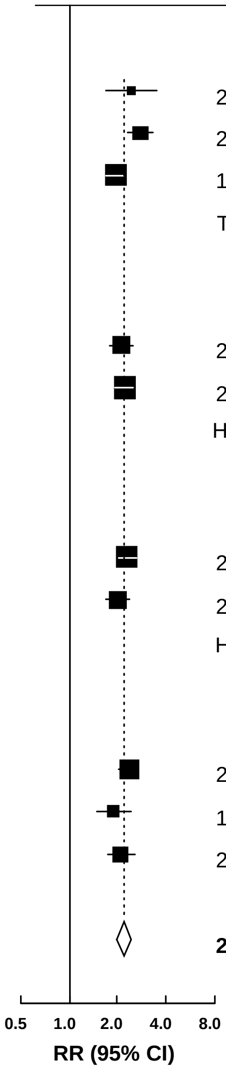

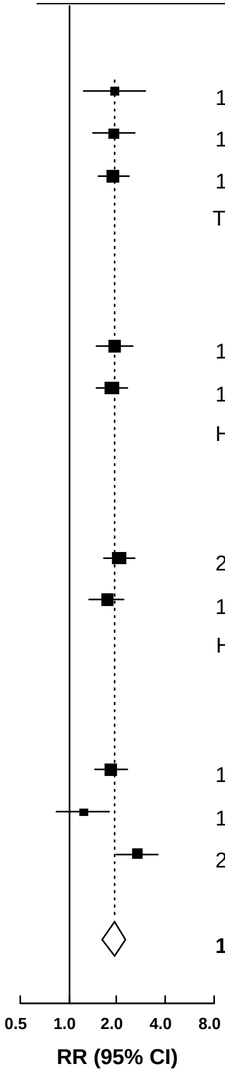

Rate ratios (RR) adjusted for age, sex, education, province, smoking, alcohol, and BMI (when appropriate), and omit the first 5 years of follow up. Exclusions as in Table 1 plus prior history of any chronic disease i.e. *vascular disease, emphysema/COPD, liver cirrhosis, chronic kidney disease, cancer or peptic ulcer. For each category, area of the square is inversely proportional to the variance of the category−specific log risk, which also determines the confidence interval (CI).*Excludes lowest category of BMI.

Figure S6. Vascular mortality vs usual systolic blood pressure (SBP) and usual BMI, at ages 35-79 years in Cuba, with further exclusion of those with non-vascular chronic disease at baseline

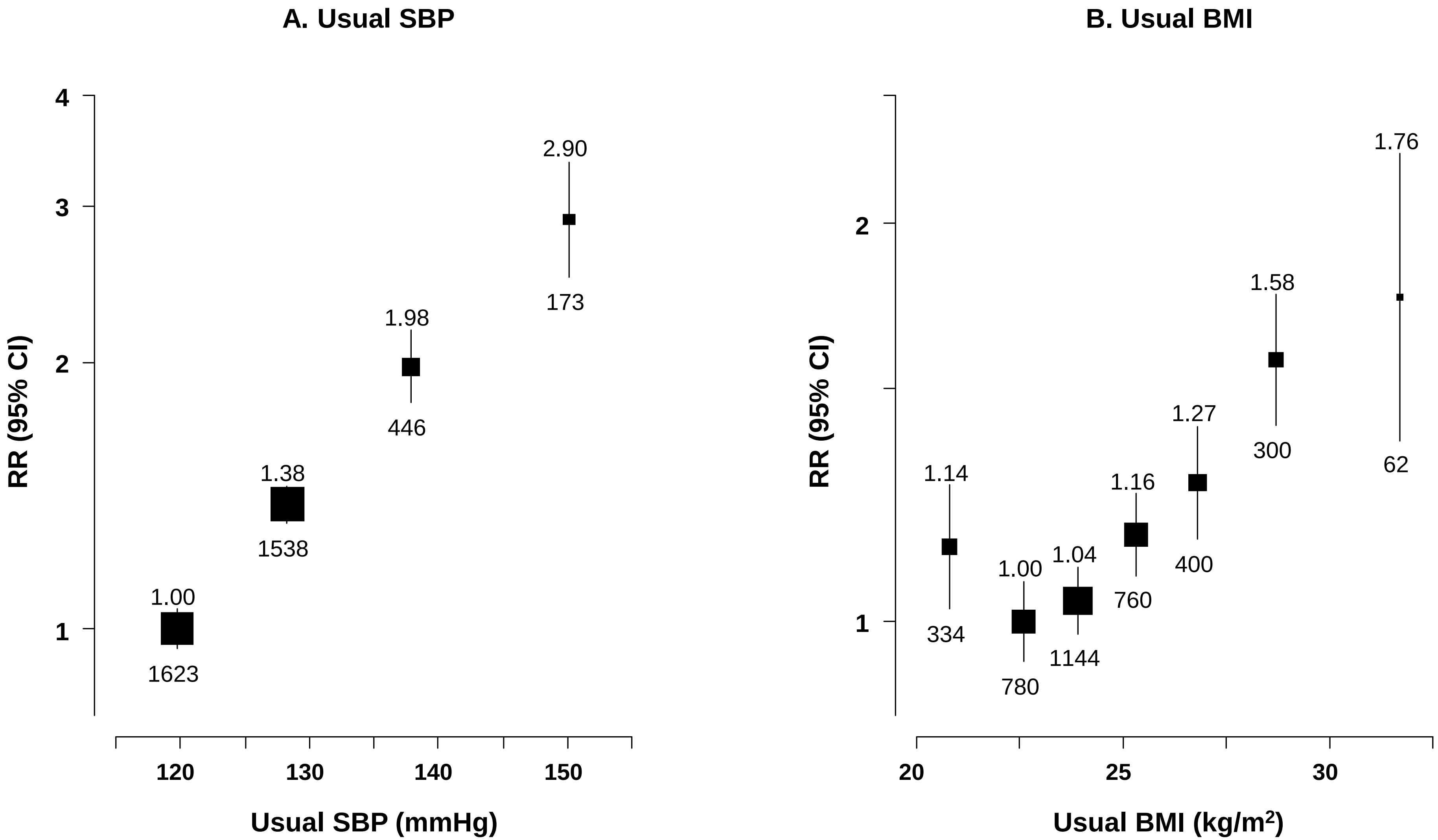

Rate ratios (RR) adjusted for age, sex, education, province, smoking, alcohol, and BMI (when appropriate), and omit the first 5 years of follow up. Exclusions as in Table 1 plus prior history of any chronic disease i.e. *vascular disease, emphysema/COPD, liver cirrhosis, chronic kidney disease, cancer or peptic ulcer. For each category, area of the square is inversely proportional to the variance of the category−specific log risk, which also determines the confidence interval (CI).

Figure S7. Death rate ratios (RR) for vascular mortality vs usual systolic blood pressure (SBP), diabetes, and usual BMI, at ages 35-79 years in Cuba, not excluding any follow-up

	Mean age at death (years)	Deaths	RR (CI) per 20 mmHg higher usual SBP	RR (CI) for diabetes vs no diabetes	RR (CI) per 10 kg/m^2 higher usual BMI*
**Age-at-risk (years)**					
35-59	54	912	3.02 (2.56 - 3.55)	2.74 (2.14 - 3.51)	1.71 (1.20 - 2.44)
60-69	66	1674	2.27 (2.03 - 2.53)	2.50 (2.15 - 2.91)	1.79 (1.39 - 2.30)
70-79	75	2965	1.67 (1.54 - 1.82)	1.93 (1.74 - 2.15)	1.66 (1.38 - 2.01)
			Trend test: χ^2_1=46.6 (p<0.001)	Trend test: χ^2_1=10.6 (p=0.001)	Trend test: χ^2_1=0.1 (p=0.8)
**Sex**					
Men	68	2924	2.05 (1.88 - 2.25)	2.06 (1.80 - 2.36)	1.75 (1.42 - 2.16)
Women	69	2627	1.92 (1.76 - 2.09)	2.21 (1.99 - 2.45)	1.65 (1.37 - 1.99)
			Heterogeneity test: χ^2_1=1.2 (p=0.3)	Heterogeneity test: χ^2_1=0.7 (p=0.4)	Heterogeneity test: χ^2_1=0.2 (p=0.7)
**Smoking**					
Never	70	2604	1.85 (1.69 - 2.02)	2.25 (2.02 - 2.51)	1.76 (1.46 - 2.14)
Ever	68	2947	2.12 (1.94 - 2.31)	2.02 (1.78 - 2.30)	1.65 (1.35 - 2.01)
			Heterogeneity test: χ^2_1=4.5 (p=0.03)	Heterogeneity test: χ^2_1=1.6 (p=0.2)	Heterogeneity test: χ^2_1=0.2 (p=0.6)
**Subtype**					
IHD	69	2729	2.05 (1.87 - 2.24)	2.35 (2.09 - 2.64)	1.77 (1.45 - 2.16)
Stroke	69	1144	2.01 (1.75 - 2.30)	1.74 (1.43 - 2.11)	1.21 (0.88 - 1.66)
Other vascular	70	1678	1.87 (1.67 - 2.09)	2.15 (1.85 - 2.50)	2.03 (1.58 - 2.60)
**Overall**	**69**	**5551**	**1.98 (1.86 - 2.11)**	**2.16 (1.99 - 2.34)**	**1.71 (1.49 - 1.97)**

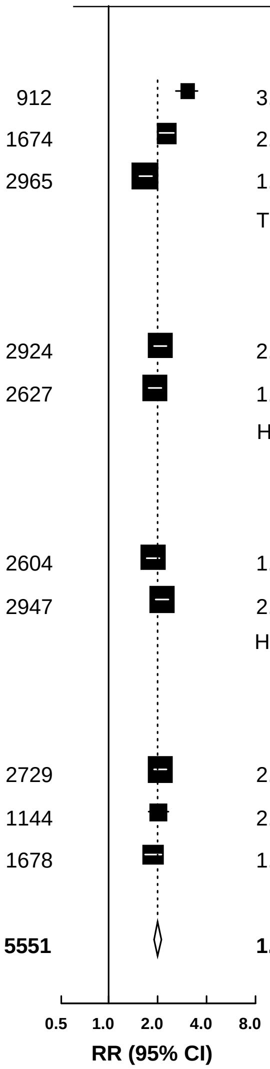

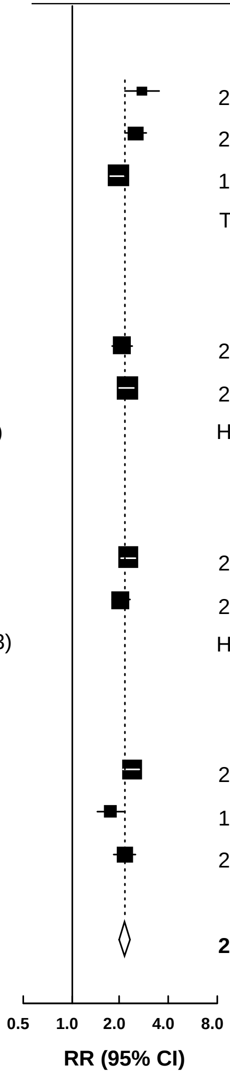

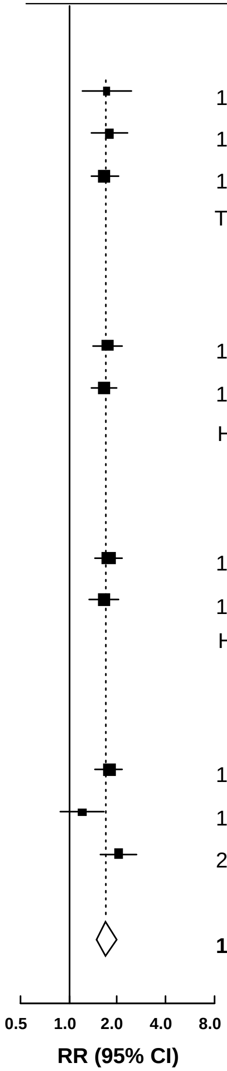

Rate ratios (RR) adjusted for age, sex, education, province, smoking, alcohol, and BMI (when appropriate), and did not omit the first 5 years of follow up. Exclusions as in Table 1. For each category, area of the square is inversely proportional to the variance of the category−specific log risk, which also determines the confidence interval (CI).*Excludes lowest category of BMI.

Figure S8. Vascular mortality vs usual systolic blood pressure (SBP) and usual BMI, at ages 35-79 years in Cuba, <u>not excluding any follow-up</u>

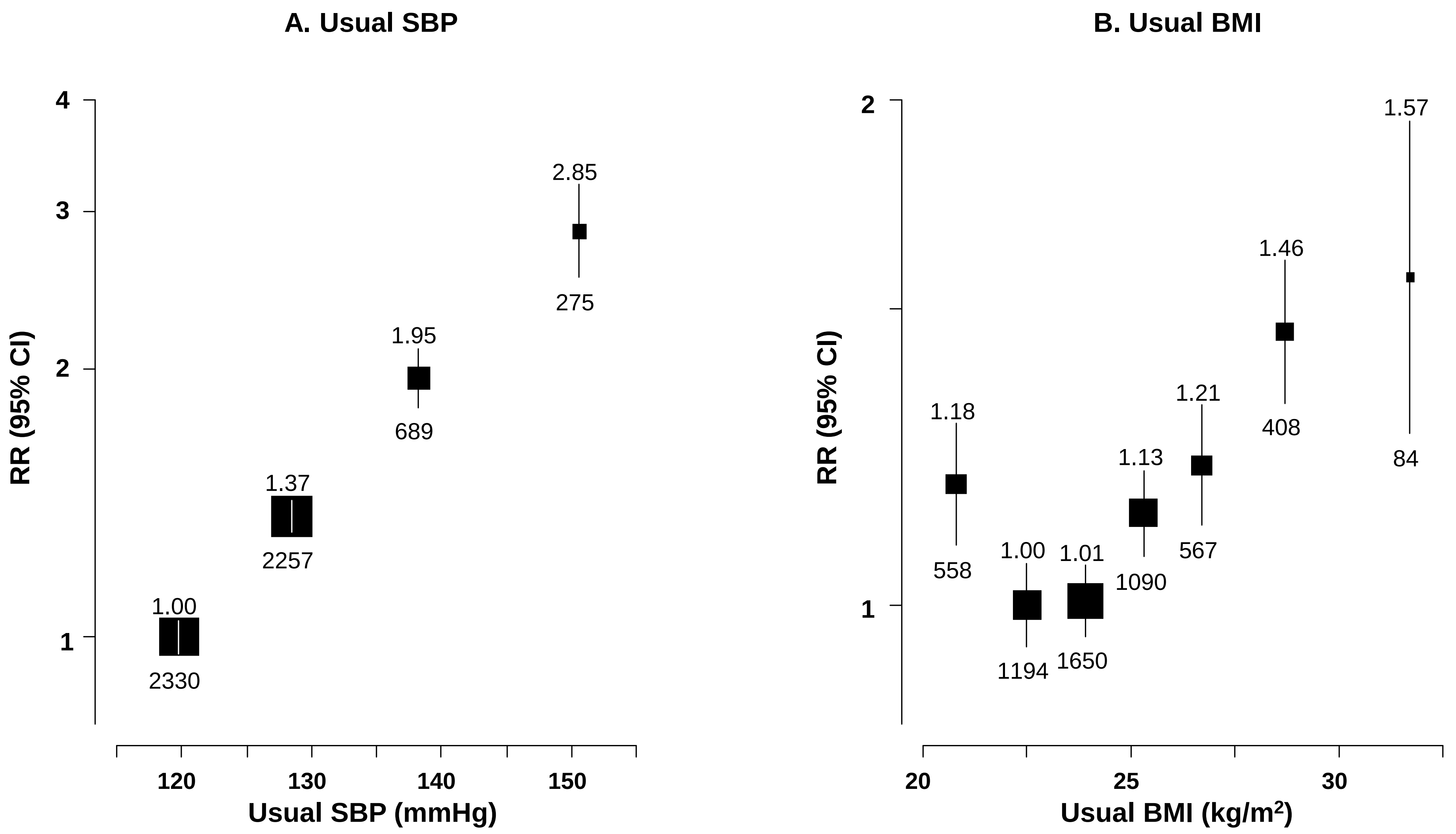

Rate ratios (RR) adjusted for age, sex, education, province, smoking, alcohol, and BMI (when appropriate), and did not omit the first 5 years of follow up. Exclusions as in Table 1. For each category, area of the square is inversely proportional to the variance of the category−specific log risk, which also determines the confidence interval (CI).

Figure S9. Death rate ratios (RR) for vascular mortality vs usual systolic blood pressure (SBP), diabetes, and usual BMI at ages 35-79 years in Cuba, excluding those taking blood pressure-lowering medication at baseline

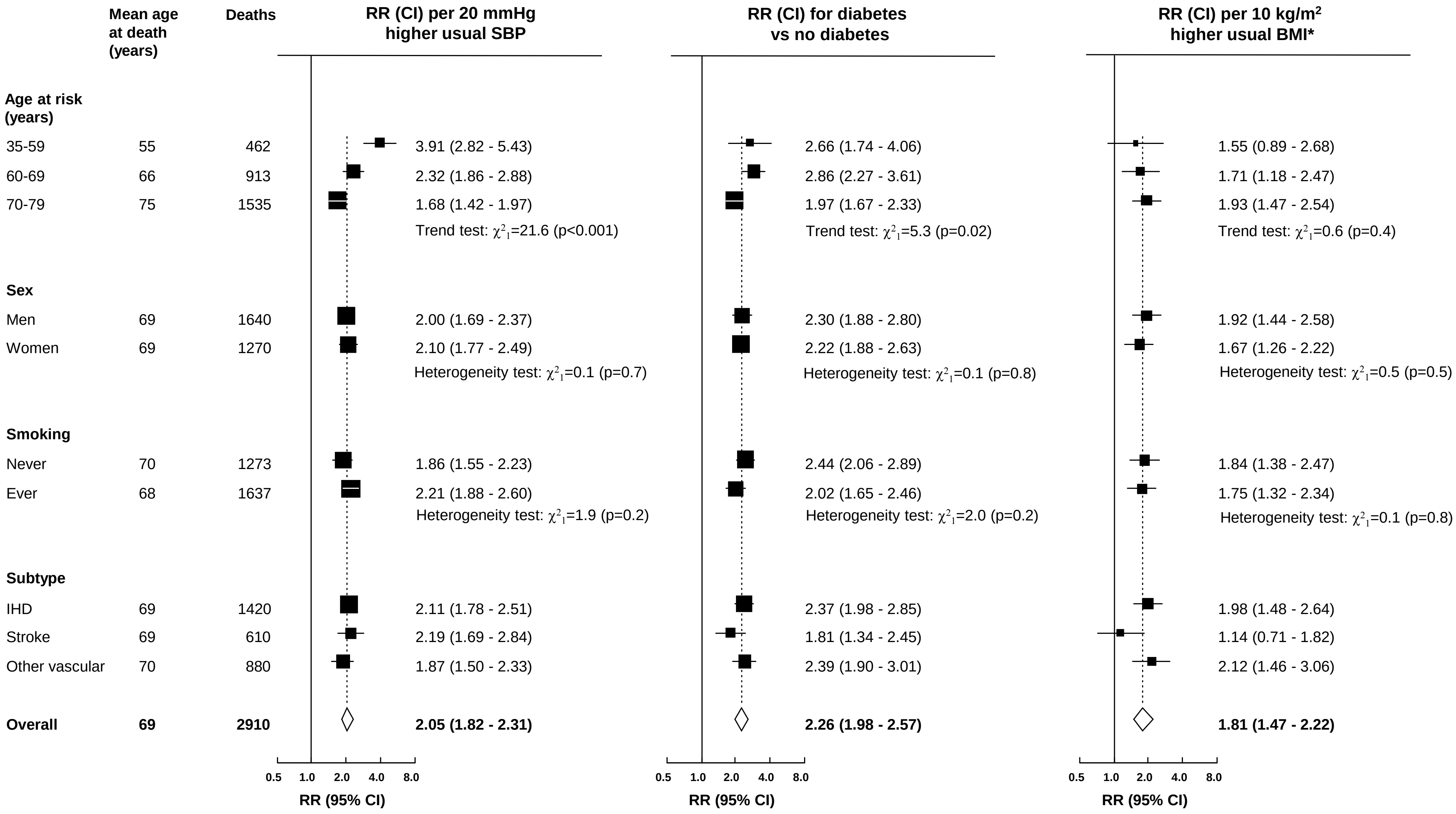

Rate ratios (RR) adjusted for age, sex, education, province, smoking, alcohol, and BMI (when appropriate), and omit the first 5 years of follow up. Exclusions as in Table 1, with further exclusion of those on blood pressure-lowering medication. For each category, area of the square is inversely proportional to the variance of the category−specific log risk, which also determines the confidence interval (CI).*Excludes lowest category of BMI.

Figure S10. Vascular mortality vs usual systolic blood pressure (SBP) and usual BMI, at ages 35-79 years in Cuba, excluding those taking blood pressure-lowering medication at baseline

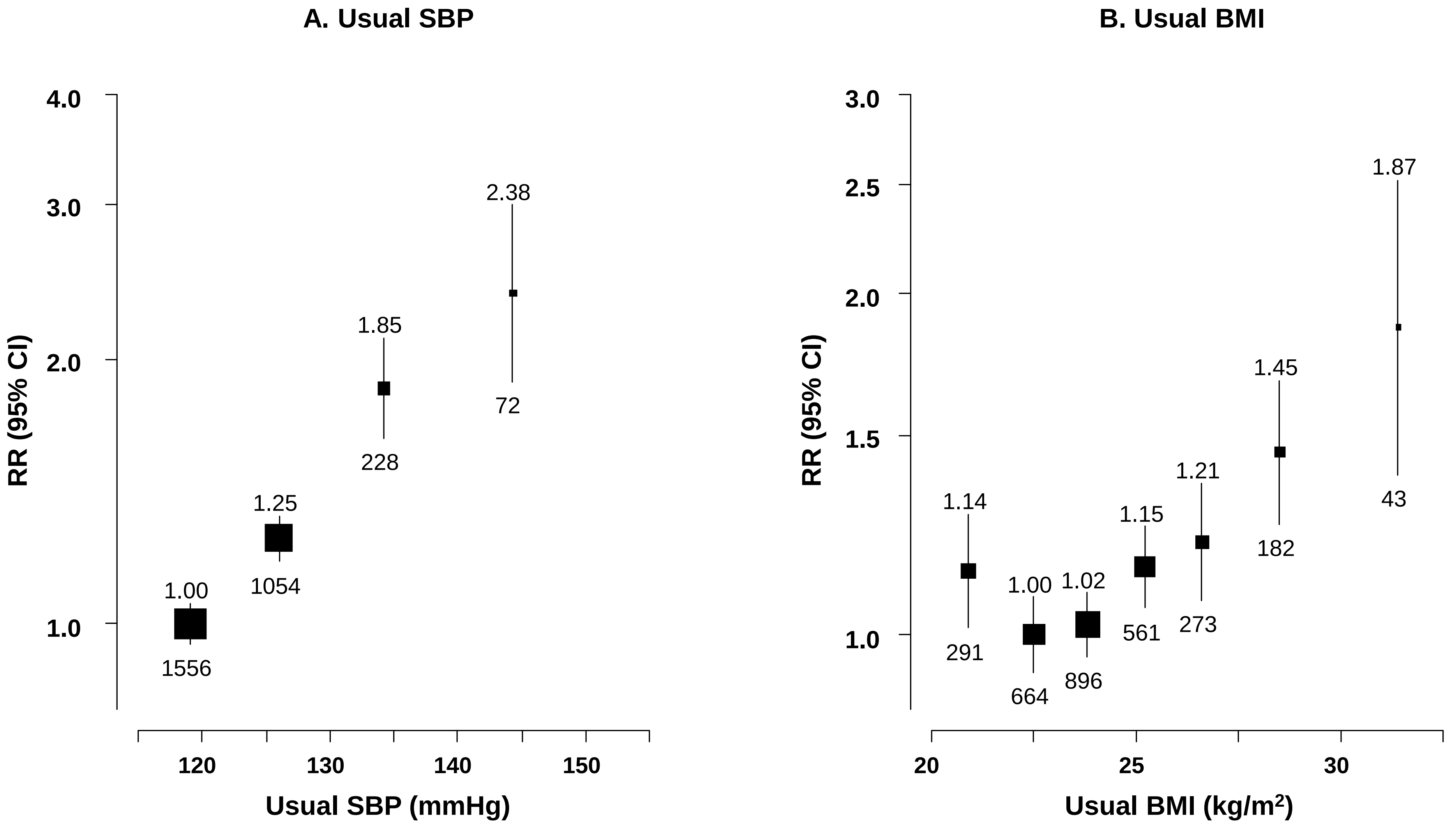

Rate ratios (RR) adjusted for age, sex, education, province, smoking, alcohol, and BMI (when appropriate), and omit the first 5 years of follow up. Exclusions as in Table 1, with further exclusion of those on blood pressure-lowering medication at baseline. For each BMI category, area of the square is inversely proportional to the variance of the category-specific log risk, which also determines the confidence interval (CI).

Figure S11. Death rate ratios (RR) for vascular mortality (excluding ischaemic heart disease and stroke) vs usual systolic blood pressure (SBP), diabetes, and usual BMI at ages 35-79 years in Cuba

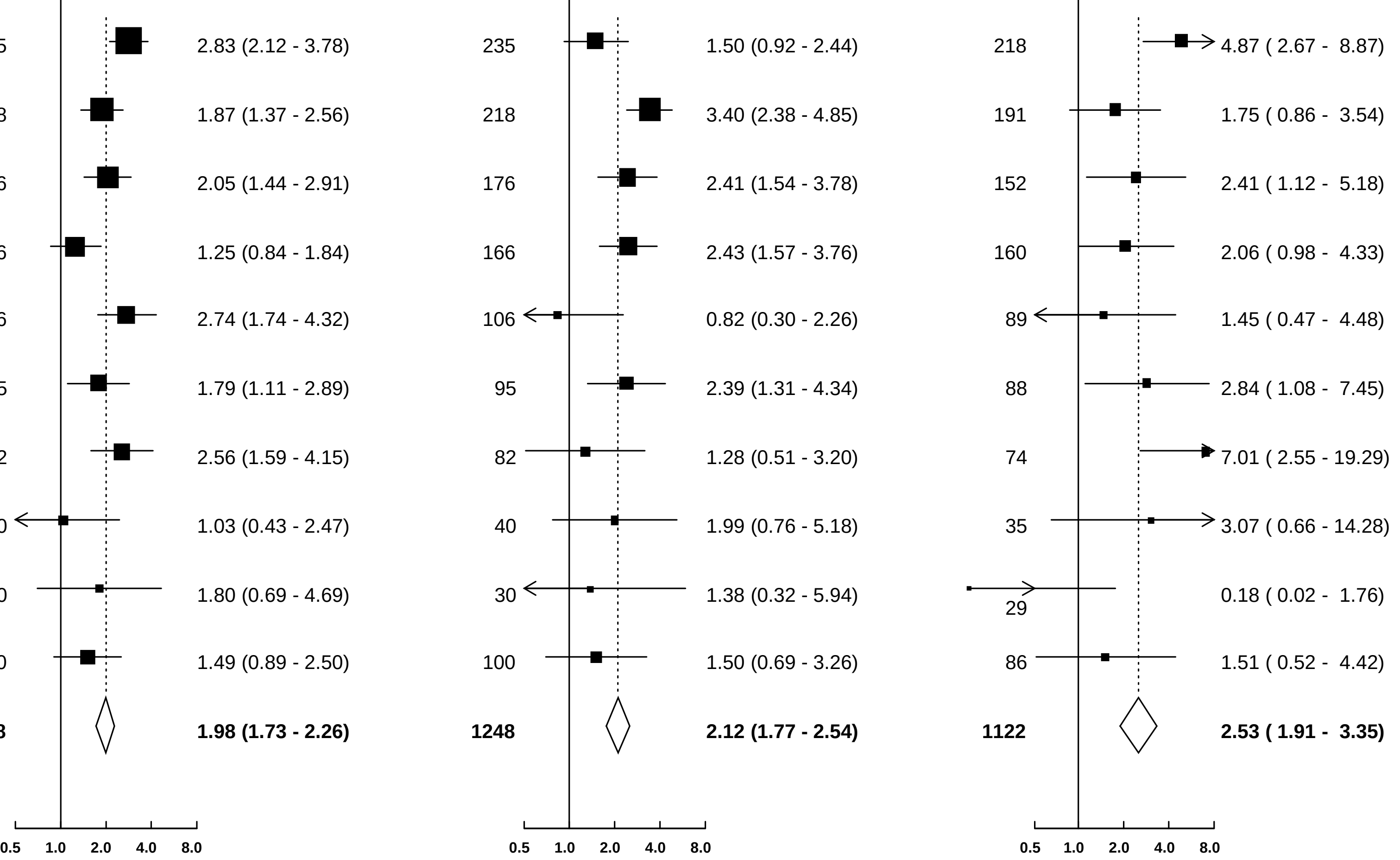

	Mean age at death (years)	SBP: Deaths	SBP: RR & CI	Diabetes: Deaths	Diabetes: RR & CI	BMI: Deaths	BMI: RR & CI
Hypertensive disease	69	235	2.83 (2.12 - 3.78)	235	1.50 (0.92 - 2.44)	218	4.87 (2.67 - 8.87)
Heart failure	72	218	1.87 (1.37 - 2.56)	218	3.40 (2.38 - 4.85)	191	1.75 (0.86 - 3.54)
Other cerebrovascular (not stroke)	72	176	2.05 (1.44 - 2.91)	176	2.41 (1.54 - 3.78)	152	2.41 (1.12 - 5.18)
Atherosclerosis	72	166	1.25 (0.84 - 1.84)	166	2.43 (1.57 - 3.76)	160	2.06 (0.98 - 4.33)
Aortic aneurysm	70	106	2.74 (1.74 - 4.32)	106	0.82 (0.30 - 2.26)	89	1.45 (0.47 - 4.48)
Cardiac arrhythmia	70	95	1.79 (1.11 - 2.89)	95	2.39 (1.31 - 4.34)	88	2.84 (1.08 - 7.45)
Inflammatory heart disease	67	82	2.56 (1.59 - 4.15)	82	1.28 (0.51 - 3.20)	74	7.01 (2.55 - 19.29)
Pulmonary embolism	67	40	1.03 (0.43 - 2.47)	40	1.99 (0.76 - 5.18)	35	3.07 (0.66 - 14.28)
Rheumatic heart disease	64	30	1.80 (0.69 - 4.69)	30	1.38 (0.32 - 5.94)	29	0.18 (0.02 - 1.76)
Other	70	100	1.49 (0.89 - 2.50)	100	1.50 (0.69 - 3.26)	86	1.51 (0.52 - 4.42)
**All non-IHD, non-stroke vascular**	**70**	**1248**	**1.98 (1.73 - 2.26)**	**1248**	**2.12 (1.77 - 2.54)**	**1122**	**2.53 (1.91 - 3.35)**

Rate ratios (RR) adjusted for age, sex, education, province, smoking, alcohol, and BMI (when appropriate). Analyses omitted the first 5 years of follow up. Exclusions as in Table 1. For each category, area of the square is inversely proportional to the variance of the category-specific log risk, which also determines the confidence interval (CI).*Excludes lowest category of BMI.
